# Taurodeoxycholate Increases the Number of Myeloid-Derived Suppressor Cells That Ameliorate Sepsis in Mice

**DOI:** 10.3389/fimmu.2018.01984

**Published:** 2018-09-18

**Authors:** Sooghee Chang, Youn-Hee Kim, Young-Joo Kim, Young-Woo Kim, Sungyoon Moon, Yong Yook Lee, Jin Sun Jung, Youngsoo Kim, Hi-Eun Jung, Tae-Joo Kim, Taek-Chin Cheong, Hye-Jung Moon, Jung-Ah Cho, Hang-Rae Kim, Dohyun Han, Yirang Na, Seung-Hyeok Seok, Nam-Hyuk Cho, Hai-Chon Lee, Eun-Hee Nam, Hyosuk Cho, Murim Choi, Nagahiro Minato, Seung-Yong Seong

**Affiliations:** ^1^Department of Microbiology and Immunology, Seoul National University College of Medicine, Seoul, South Korea; ^2^Department of Biomedical Sciences, Seoul National University College of Medicine, Seoul, South Korea; ^3^Wide River Institute of Immunology, Seoul National University, Seoul, South Korea; ^4^Department of Anatomy, Seoul National University College of Medicine, Seoul, South Korea; ^5^Biomedical Research Institute, Seoul National University Hospital, Seoul, South Korea; ^6^Department of Immunology and Cell Biology, Graduate School of Medicine, Kyoto University, Kyoto, Japan

**Keywords:** sepsis, myeloid-derived suppressor cells, taurodeoxycholate, TGR5, inflammation

## Abstract

Bile acids (BAs) control metabolism and inflammation by interacting with several receptors. Here, we report that intravenous infusion of taurodeoxycholate (TDCA) decreases serum pro-inflammatory cytokines, normalizes hypotension, protects against renal injury, and prolongs mouse survival during sepsis. TDCA increases the number of granulocytic myeloid-derived suppressor cells (MDSC_LT_) distinctive from MDSCs obtained without TDCA treatment (MDSC_L_) in the spleen of septic mice. FACS-sorted MDSC_LT_ cells suppress T-cell proliferation and confer protection against sepsis when adoptively transferred better than MDSC_L_. Proteogenomic analysis indicated that TDCA controls chromatin silencing, alternative splicing, and translation of the immune proteome of MDSC_LT_, which increases the expression of anti-inflammatory molecules such as oncostatin, lactoferrin and CD244. TDCA also decreases the expression of pro-inflammatory molecules such as neutrophil elastase. These findings suggest that TDCA globally edits the proteome to increase the number of MDSC_LT_ cells and affect their immune-regulatory functions to resolve systemic inflammation during sepsis.

## Introduction

Bile acids (BAs) are amphiphilic surfactant molecules synthesized from cholesterol in the liver (1). There are various BAs in mammals (2). Cholic acid (CA) and Chenodeoxycholic acid (CDCA) are primary BAs, and CDCA gives rise to taurochenodeoxycholate (TCDC) and glycochoenodeoxycholate (GCDC) after conjugation with taurine and glycine, respectively (1). Intestinal bacteria produce secondary BAs, such as lithocholic acids (LCA) and deoxycholic acid (DCA), from primary BAs (1). The diversity of the BA pool is further increased following modification by various liver enzymes during the enterohepatic circulation of BAs (1).

BAs in millimolar concentrations play roles in solubilizing fat-soluble nutrients to facilitate their gastrointestinal uptake (3). In addition, BAs play roles as signaling molecules that activate several receptors at micromolar concentrations (4). The diverse metabolic functions of BAs are a result of their interaction with various receptors of which the specificities are determined by 3-, 7-, 12-hydroxyl groups and taurine- or glycine-conjugation of BAs (4). BAs interact with nuclear receptors, such as steroid and xenobiotic receptor (SXR), constitutive androstane receptor (CAR), pregnane X receptor (PXR), vitamin D receptor (VDR) and farnesoid X receptor (FXR), and cell-surface membrane receptors, such as Takeda G protein-coupled receptor 5 (TGR5), sphingosine-1- phosphate receptor 2 (S1PR2), α1β4/α5β1 integrin, and muscarinic acetylcholine receptors (mAChRs), as well as large conductance Ca^2+^-activated K^+^ channels (3–6).

BAs modulate inflammation (4) in addition to metabolic homeostasis. For example, CDCA and CA bind with FXR and regulate inflammatory diseases (7). CDCA and DCA inhibit LPS-induced TNF secretion (8). Formyl-peptide receptor (FPR)-mediated chemotaxis of leukocytes is also inhibited by CDCA interaction with mAChRs (9). LCA inhibits NLRP3 inflammasome activation via the TGR5 (10). Conjugated bile acids also activate TGR5 to regulate inflammation (8–11). However, the exact mechanism of immune modulation by BAs is promiscuous because a BA interacts with multiple receptors and a BA receptor interacts with different types of BAs (2). For example, LCA binds with FXR (12), PXR (13), SXR (14), and VDR (15), as well as membrane TGR5 (10). TGR5 and FXR are activated by ursodeoxycholic acid (UDCA), DCA, CDCA, and LCA (16, 17).

In addition to the complex interaction between BAs and receptors, the promiscuity of BA-mediated immune regulation mechanisms has increased because many studies were conducted using BAs at concentrations that are not attainable under pharmacological conditions or pathological conditions *in vivo*. For example, 10~30 μM of LCA was necessary to inhibit activation of the NLRP3 inflammasome (10). However, the median plasma level of LCA in healthy individuals and in pathological conditions is 10 ~ 35 nM (18), which suggests that approximately 1,000~3,000 × the physiological or pathological concentration is necessary to inhibit NLRP3 activation by LCA. Furthermore, whether this high plasma concentration could be attainable pharmacologically *in vivo* has not been investigated.

Among the BA receptors, TGR5 has received substantial attention because of the many studies that suggest the crucial roles of TGR5 in immune regulation (19). For example, various TGR5 agonists inhibit inflammation of the stomach (20) and brain (21). Functional impairment of TGR5 incurs more severe inflammation than wild-type mice in response to LPS (22) and contributes to autoimmune diseases (23). TGR5 agonists also negatively modulate NF-κB (24), and the TGR5-AKT-mTOR1 pathway inhibits macrophage chemotaxis (25).

In this study, we used taurodeoxycholic acid (TDCA) to investigate the mechanism of immune modulation rather than other BAs because taurine-conjugated BAs activate the TGR5 pathway better than unconjugated BAs and glycine-conjugated BAs (26, 27). In addition, taurine-conjugated BAs exhibit less cytotoxicity than unconjugated BAs and glycine-conjugated BAs (28). TLCA exhibited a lower EC50 in TGR5 pathway activation; however, TLCA is more cytotoxic than TDCA (27, 29). For this reason, we evaluated the mode of immune regulation by TDCA, which activates the TGR5 pathway (30).

In this study, TDCA increased the number of CD11b^+^Gr1^hi^ granulocytic myeloid-derived suppressor cells (gMDSCs) at a pharmacologically attainable plasma concentration, which were proteogenomically different from gMDSCs obtained from septic mice without TDCA treatment and ameliorated systemic inflammation (26).

## Materials and methods

### Reagents and cells

TDCA was purchased from New Zealand Pharmaceuticals Ltd. (Palmerston North, New Zealand). LPS from *Salmonella enterica* serotype enteritidis was obtained from Sigma-Aldrich (St. Louis, MO). Fetal bovine serum, L-glutamine and 2-mercaptoethanol, penicillin, streptomycin and gentamicin were obtained from GibcoBRL (Waltham, MA). RPMI was obtained from Welgene (Gyeongsan-si, Korea). Mouse B-cell and T-cell isolation kits were obtained from Miltenyi Biotec for MACS (Bergisch Gladbach, Germany). IL-10-producing MICK-2 cells were obtained from BD Biosciences (San Jose, CA) and were used as positive controls for the FACS analysis of IL-10.

### Mice

C57BL/6N mice (B6, Shizuoka, Japan), C57BL/6-IL10^tm1Cgn^ mice (IL-10KO, The Jackson Laboratories, Bar Harbor, ME) and C57BL/6-Gpbar1^tm1(KOMP)Vlcg^ mice (TGR5 KO, KOMP Repository, The Knockout Mouse Project, University of California, Davis, CA) were housed in the Seoul National University animal facility in a specific pathogen-free environment. Eight- to Twelve-week-old female mice were used for the experiments. The Institutional Animal Care and Use Committee (IACUC) of the Biomedical Research Institute in Seoul National University Hospital (AAALAC) approved all animal experiments (SNU 10-0331). The mice were monitored every 24 h for survival and other clinical signs (ruffled fur, diarrhea, lethargy, and loss of body weight) for 14 day after sepsis induction.

### LPS injection model of sepsis

The survival rate of the female mice was determined after i.p. injection of LPS (20 mg/kg), followed by the i.v. infusion of 200 μl of PBS or TDCA for 20 min (0.5 mg/kg, unless otherwise indicated) using a Medfusion 2001 system (Medex, Dublin, OH) at 30 min (unless otherwise indicated) after LPS injection. For the protection assay using IL-10 KO mice, 5 mg/kg LPS were injected i.p. For the adoptive transfer experiments, B6 mice were injected i.v. with 100 μl of purified cells. The mice were treated with LPS 24 h prior to adoptive transfer, unless otherwise specified.

### CLP-induced sepsis model

Female B6 mice were anesthetized, and a small abdominal midline incision was made. The cecum was ligated below the ileocecal valve and punctured 3 times using a 23-gauge needle. The abdominal incision was closed with an auto-metal clip (Mikron Precision, Inc., Ontario, Canada). The same procedure was applied to the sham-operated animals, with the exception of the ligation and puncture of the cecum. The mice were subsequently infused with 200 μl of PBS or TDCA i.v. at 2 h after CLP.

### Hematoxylin and eosin staining

The tissues were fixed in 10% neutral buffered formalin solution (Sigma-Aldrich, St. Louis, MO) at room temperature (RT) for no less than 2 weeks and embedded in paraffin. The sections were stained with hematoxylin and eosin.

### PAS staining

Tissue sections in paraffin were deparaffinized using xylene for 10 min 4 times and were subsequently washed with distilled water for 5 min, followed by oxidization in 0.5% periodic acid solution for 15 min. After rinsing with distilled water, the sample was placed in Schiff reagent for 30 min and washed with running water for 5 min, followed by counterstaining with Harris hematoxylin for 5 min. After briefly washing with distilled water, the sample was dehydrated with 1% HCl alcohol for 10 min and cleared for microscopic observation.

### Blood chemistry

Mouse blood was collected at 48 h after LPS injection, and serum samples were harvested via centrifugation (2,000 × *g*, 30 min) following incubation of the blood at RT for 30 min. Alanine amino transferase (ALT), aspartate aminotransferase (AST), blood urea nitrogen (BUN) and creatinine were measured using an automatic chemistry analyzer (Hitachi 7070, Hitachi, Tokyo, Japan).

### Blood pressure

The blood pressure was determined using the tail-cuff method with a 6-channel CODA High-Throughput NIBP Acquisition system (Kent Scientific Corporation, Torrington, CT). All measurements were recorded at each time point and averaged.

### Cytokine measurements with the cytometric bead array (CBA)

Blood samples were collected in heparin-treated tubes (Sarstedt, Niimbrecht, Germany) at the indicated times after sepsis induction. The cytokine concentrations in the sera were determined using CBA according to the manufacturer's instructions (BD Biosciences). The concentrations of cytokines were calculated by a regression curve determined by known amounts of provided standard cytokines.

### FACS

Leukocyte subpopulations from the spleen and bone marrow were analyzed using a FACS Canto II (BD Bioscience) and Flowjo software (Treestar, Ashland, OR) after antibody staining. The absolute numbers of spleen cells were counted by Trypan blue exclusion, and the number of CD11b^+^Gr1^hi^ cells was calculated based on the % of total splenocytes after FACS analysis.

Splenocytes were purified at 24, 48, and 72 h after LPS injection or after CLP. Briefly, single-cell suspensions were prepared after grinding the spleen with frosted slides or flushing the bone marrow of the femur with PBS. Single-cell suspensions were filtered through a cell strainer with a 70-μm nylon mesh (BD Bioscience). After depleting red blood cells (RBCs) using ACK lysis buffer (0.15 M NH_4_Cl, 0.1 mM EDTA, 1 mM Tris pH 7.4), the splenocytes or bone marrow cells were washed with ice-cold FACS buffer (PBS that contained 1% BSA, 0.1% NaN_3_ and 1 mM EDTA) and blocked on ice for 30 min with blocking buffer [anti-FcγRIIb antibody (clone 2.4G2, BD) and 10% heat-inactivated mixed serum from mouse, rat and goat]. The cells were stained with fluorochrome-conjugated monoclonal antibodies (mAbs) against mouse CD11b (clone M1/70), Gr1 (clone RB6-8C5), Ly6c (clone ER-MP20, Abcam, Cambridge, MA), Ly6g (clone 1A8), F4/80 (clone CI:A3-1, BioLegend, San Diego, CA) and CD244 (clone eBio244F4, eBioscience). The fluorochrome-conjugated mAbs were purchased from BD Biosciences, unless otherwise specified.

To enumerate splenic T cells, the mice were infused with TDCA (or PBS) i.v. via the tail vein at 30 min after i.p. LPS (or PBS) injection. Splenocytes were harvested at 6, 24, or 48 h after LPS injection and stained with anti-CD4, anti-CD8 and anti-CD25 antibodies, followed by intracellular staining with Abs reactive with CTLA4 or Foxp3. The staining with anti-FoxP3 or anti-CTLA4 antibody was performed according to the manufacturer's protocol (Foxp3 Fixation/Permeabilization Concentrate and Diluent kit, eBioscience). The cells were surface-stained with FITC-conjugated anti-CD4 mAb and Percp Cy5.5-conjugated anti-CD25 mAb, fixed, and permeabilized for subsequent intracellular staining. For cytoplasmic staining, the permeabilized cells were blocked on ice for 30 min with blocking buffer and stained using PECy7-conjugated anti-FoxP3 and PE-conjugated anti-CTLA4 mAbs. The cells were stained with 7-amino actinomycin D (7AAD) (BD Bioscience) to determine the cell viability.

IL-10 production by the cells was determined using FACS. Splenocytes were obtained from 4 groups of mice: the PBS + PBS group, PBS + TDCA group, LPS + PBS group or LPS + TDCA group. The splenocytes were stimulated with phorbol 12-myristate 13-acetate (50 ng/ml, Sigma) plus ionomycin (1 μg/ml, Sigma) for 4 h. The splenocytes were stained with Abs against CD11b and Gr1, followed by cytoplasmic staining with Abs against IL-10 after permeabilizing the cells with a one-step BD Cytofix/Cytoperm kit (BD Biosciences). Isotype control antibodies were included in all staining sets to evaluate nonspecific antibody binding.

### T-cell proliferation assay

T cells were purified from mouse spleen using MACS with a pan T-cell isolation kit (Miltenyi Biotec). A total of 2 × 10^5^ normal splenic T cells were stimulated with 1 μg/ml of anti-CD3 (eBioscience, Waltham, MA) and 10 μg/ml of anti-CD28 (BD Bioscience) antibodies in RPMI 1640 medium containing 10% heat-inactivated FBS and 2 mM glutamine. FACS-sorted CD11b^+^Gr1^hi^ cells from the LPS + PBS group or the LPS + TDCA group were mixed with T cells at a final concentration of 4 × 10^4^/well (E:T = 1:5) in a 96-well flat-bottom plate (Nunc, Roskilde, Denmark) and cultured for 96 h at 37°C in a humidified 5% CO_2_ atmosphere. T cells cultured without CD11b^+^Gr1^hi^ cells served as a negative control. The cells were pulsed with 1 μCi [^3^H] methyl-thymidine (Perkin Elmer, Waltham, MA) for 18 h. The cells were harvested with a Filtermate Harvester (Perkin Elmer), and the isotope incorporation was measured using a MicroBeta Plate Counter (Perkin Elmer). The data are expressed as the counts per minute (cpm) ± standard error of the mean (SEM).

### Adoptive transfer of CD11b^+^Gr1^hi^ cells

PBS or TDCA was infused i.v. via the tail vein at 30 min after LPS injection i.p. into B6 mice. The cells were isolated from the spleen at 24 h after LPS injection. Following incubation of the cells in blocking buffer for 30 min on ice, the cells were stained with a biotin-conjugated anti-mouse Gr1 antibody (clone RB6-8C5), followed by staining with anti-biotin microbeads (Miltenyi Biotec) at 4°C for 15 min. After washing with MACS buffer (0.5% BSA and 2 mM EDTA in PBS), the cells were positively selected using an LS column (Miltenyi Biotec). The Gr1^+^ cells presorted with MACS were further stained with mAbs against CD11b (conjugated to PE) and F4/80 (conjugated to FITC) for FACS sorting. The CD11b^+^Gr1^hi^ F4/80^int^ cells were sorted using a FACSAria (BD Bioscience). A total of 1 × 10^5^ cells were injected i.v. via the tail vein into B6 mice. The recipient mice were injected i.p. with LPS 24 h prior to adoptive transfer.

### Microarray and RT-PCR

CD11b^+^Gr1^hi^ cells were purified by FACS. Total RNA was extracted from the cells using an RNeasy Mini kit according to the manufacturer's instructions (QIAGEN). The differential expression was assessed using a GeneChip Scanner 3000 7G (Affymetrix, Mouse 430_2, genome version mm10). A total of 14,074 genes (*p* < 0.05) were filtered, and 818 genes that exhibited >8-fold changes (*p* < 0.05) were analyzed using DNASTAR® (DNASTAR Inc.). A hierarchical clustering method (distance metric by Euclidean) was used to generate heatmaps. Genes with corrected *p-*values < 0.05 (one-way ANOVA, unpaired) and fold changes >2 or <-2 were considered significantly regulated. The microarray data have been deposited in the Gene Expression Omnibus under accession number GSE92948. For RT-PCR analysis, total RNA was isolated as previously described. cDNA was synthesized with an Omniscript RT KIT (QIAGEN) and PCR-amplified (MyCycler™ Thermal Cycler, BIO-RAD, Hercules, CA) using the Plantinum® PCR SuperMix (Thermo Fisher Scientific, Bremen, Germany). The primer sequences and PCR conditions are summarized in Table S1. Ingenuity Pathway Analysis (IPA®, QIAGEN, Redwood City) was used for the pathway analysis.

### In-solution digestion of cell lysates

The CD11b^+^Gr1^hi^ cells were FACS-sorted from 2 groups of mice (LPS+PBS and LPS+TDCA). The lysates of the FACS-sorted CD11b^+^Gr1^hi^ splenocytes were prepared using 8 M urea buffer, and the protein concentrations were determined by the BCA assay (Micro BCA Protein Assay Kit, ThermoFisher Scientific, Bremen, Germany). Dithiothreitol was added to the lysate (3 mM) and incubated at room temperature for 1 h. The cell lysates were mixed with iodoacetamide (5 mM) and incubated in a dark room for 1 h. One part of the lysates was mixed with 10 parts of 50 mM ammonium bicarbonate and digested with trypsin (1/50 × total protein amount of cell lysate, Promega, Madison, WI) at 37°C for 16 h. The samples were subsequently desalted using a Macro Spin Column (C-18; Harvard Apparatus, Holliston, MA). The column was activated with 0.1% trifluoroacetic acid (TFA) in 80% acetonitrile in advance and subsequently equilibrated with 0.1% TFA in water (pH < 3.0). The samples were loaded into the column and centrifuged at 1,000 × *g* for 2 min at room temperature. The column was washed with 0.1% TFA in water, and the peptide fraction was eluted with 0.1% TFA in 80% acetonitrile. The peptide samples were dried with a CentriVap® benchtop vacuum concentrator (Labconco, Kansas City, MO), and the peptide concentration was determined using a BCA kit.

### Labeling peptides for iTRAQ

Isobaric tags for relative and absolute quantitation (iTRAQ) were used to compare the proteomes of CD11b^+^GR1^hi^ cells from 2 groups (LPS + PBS and LPS + TDCA). One hundred micrograms of peptide from each group were labeled according to the manufacturer's protocol for the iTRAQ reagent kit (Sciex, MA). Briefly, the peptide samples were reconstituted with 500 mM triethylammonium bicarbonate (TEAB) buffer, sonicated and vortexed. The 4-plex iTRAQ reagent dissolved with ethanol was added to the peptide samples and incubated at room temperature for 1 h. To one part of the sample, 3 parts of 0.05% TFA was added and incubated at room temperature for 30 min. The iTRAQ reagent-labeled peptides were pooled and subsequently concentrated to 300 μl using a CentriVap® benchtop vacuum concentrator (Labconco). The samples were then mixed with 1 ml of 50 mM triethylammonium bicarbonate (TEAB).

### High pH reversed-phase fractionation

The labeled peptides were separated via high pH reversed-phase fractionation using an Agilent 1260 HPLC infinity purification system (Agilent Technology, Santa Clara, CA). Briefly, an Xbridge C-18 column (ZORBAX, 4.6 × 250 mm, 5 μm, 300 Å; Waters, Milford, MA) was equilibrated with 10 mM ammonium formate in water. Seven hundred micrograms of an iTRAQ-labeled peptide sample were loaded onto the column. The peptides were serially fractionated with 10 mM ammonium formate in acetonitrile (15, 28.5, 34, 60% acetonitrile), and 8 elution fractions were separately collected. The elution samples were dried in a Centrivap (Labconco). The dried peptides were reconstituted with resolution buffer (0.1% formic acid).

### Q exactive™ hybrid quadrupole-orbitrap™ mass spectrometry

The fractionated peptide samples were subsequently loaded onto trap (C18, 3 μm, 0.7 cm, Thermo Fisher Scientific) and EASY-Spray columns (C18, 2 μm, 100 Å, 50 cm, Thermo Fisher Scientific). Easy nano II Ultra Performance Liquid Chromatography and Q-Exactive Mass Spectrometry systems (Thermo Fisher Scientific) were used to separate the peptides. The peptides were separated at a flow rate of 250 nl/min with a gradient (2–35% acetonitrile in 0.1% formic acid) for 65 min, followed by washing with 90% acetonitrile in 0.1% formic acid for 10 min. For Q-Exactive, a top 10 method was used. The Orbitrap mass analyzer was used to acquire full MS scans (m/z 300–1,600 range; resolution, 70,000). The AGC target value was 3.0E + 6. The ten most intense peaks with charge states ≥2 were fragmented in the HCD collision cell (normalized collision energy of 32%). The tandem mass spectrum was acquired in the Orbitrap mass analyzer (resolution, 17,500; AGC target value, 1.0E + 5; the intensity threshold, 8.3E + 3; the maximum allowed ion accumulation times, 20 ms for full MS scans and 120 ms for tandem mass spectrum). The peaks with 1 and 5 more charged states were excluded. The same precursor ions were also excluded after 20 s using the dynamic exclusion function.

### Annotation of peptide sequences

The Trans Proteomic Pipeline (Seattle Proteomic Center, Seattle, WA, USA) was used to convert the mass data files into mzXML files. Peptide masses were searched using a concatenated forward and reverse mouse international protein index (IPI) database (decoy ipi.MOUSE.v3.80 database, 54285 entries) (31) with the SEQUEST-Sorcerer platform (Thermo Fisher Scientific, Sage-N Research, Milpitas, CA). Sorcerer (Sage-N Research, Milpitas, CA) was used to estimate the false discovery rate (FDR). All searches were performed based on the trypsin specificity, allowing two missed cleavages. Carbamidomethylation of cysteine was set as a fixed modification, and the oxidation of methionine, N-term and lysine iTRAQ modifications were set as variable modifications. The precursor ion mass tolerance and the fragment ion mass tolerance were set to 10 ppm and 1.0 Da, respectively.

Scaffold Q+ (Proteome Software, Portland, OR) was used to compare spectral counts, validate MS/MS-based peptides, identify proteins (FDR < 1% in at least 2 peptides), and calculate log2-fold changes (FC) and *p-*values (Student's *t*-test). Proteins with redundant peptides and multiple isoforms that could not be differentiated based on MS/MS spectra were grouped separately (= primarily assigned protein). Differentially expressed proteins (FC > 1.5) were analyzed using IPA®.

### MPO and NE activity

The myeloperoxidase (MPO) activity of the lysed CD11b^+^Gr1^hi^ cells was determined using a fluorometric MPO activity assay kit (Abcam, Cambridge, MA) according to the manufacturer's protocol. The fluorescent signal was detected with a multi-detection microplate reader (Cytation 3, Biotek Instruments, Winooski, VT, USA). The NE activity of the lysed CD11b^+^Gr1^hi^ cells was determined using a mouse neutrophil elastase (NE) activity ELISA kit (Cusabio Corporation, Wuhan, China) according to the manufacturer's instructions.

### Antibody-mediated neutralization assay

At 1, 24, and 48 h after LPS injection i.p., 150 μg of anti-CD244 antibody (clone eBio244F4) or rat IgG2a isotype control antibody was injected i.p. into B6 mice together with an i.v. TDCA infusion. In addition, 100 μg of anti-CD244 antibody was injected i.p. into recipient B6 mice at 30 min and 24 h after the adoptive transfer of 1 × 10^5^ CD11b^+^Gr1^hi^ cells.

### Statistical analysis

The data are expressed as the mean ± SEM. Student's two-tailed *t*-tests were employed to compare the test group with the control group, unless otherwise indicated. A Kaplan-Meier survival analysis and log-rank test were used to calculate the mean survival time and determine the statistical significance of the survival differences. *P* < 0.05 was considered statistically significant.

## Results

### TDCA confers protection to mice with sepsis

**Figure 1:**
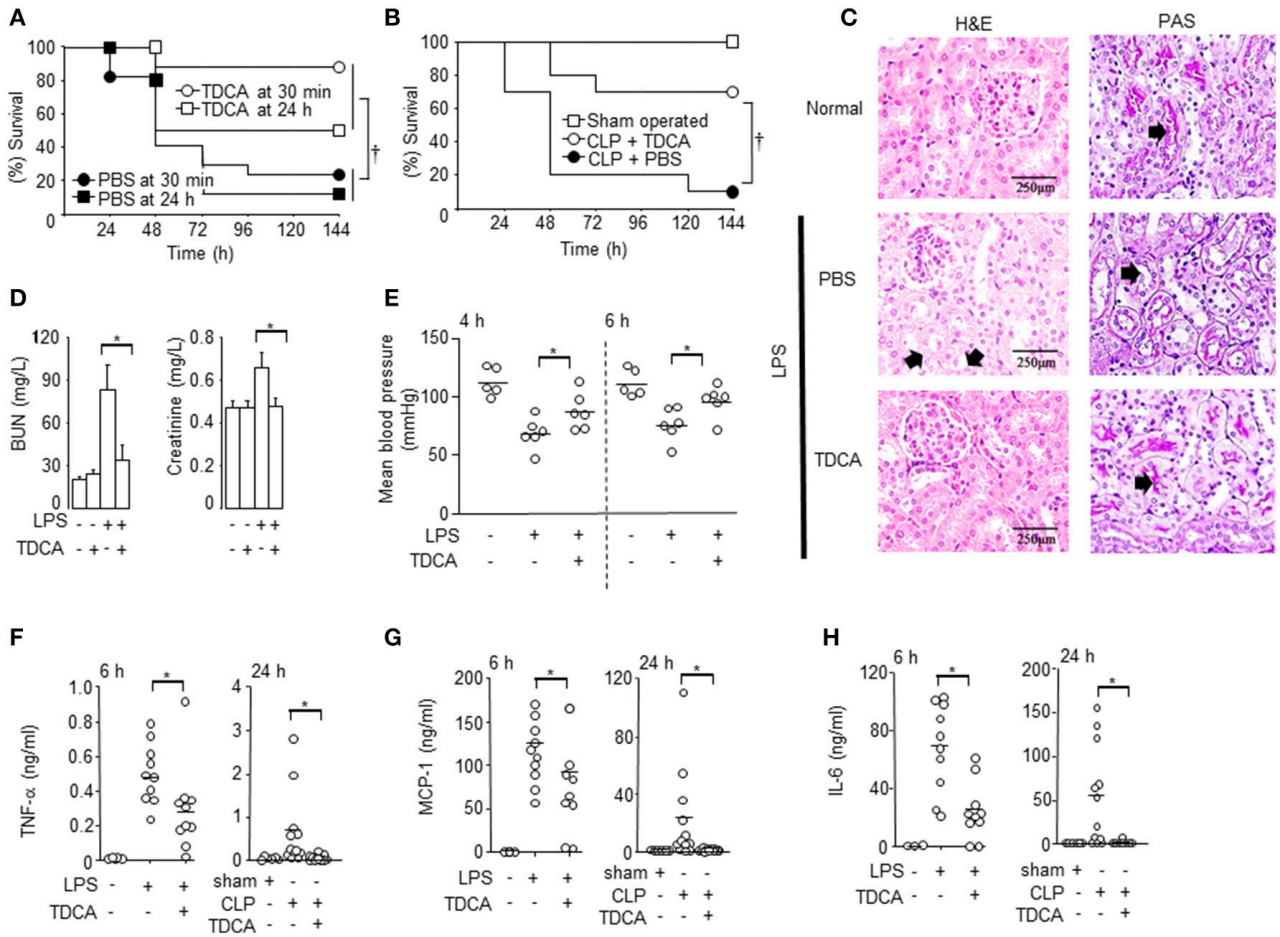
Protection of mice with sepsis after TDCA treatment. **(A)** The survival of mice under sepsis when TDCA or PBS was administered i.v. at 30 min or 24 h after LPS injection. *n* = 17 for 30 min and *n* = 8 for 24 h **(B)** The survival of mice after i.v. infusion of TDCA (°, *n* = 10) or PBS (•, *n* = 10) at 2 h after cecal-ligation and puncture. Sham group (□, *n* = 5). **(C)** H&E staining (left column) and PAS staining (right column) of kidney from mice with sepsis at 48 h after treatment with TDCA (*n* = 6) or PBS (*n* = 5). *n* = 3 for the normal B6 mice. Red arrows in the left column and right column denote representative vacuolar degeneration and loss of the brush border, respectively. **(D)** BUN and creatinine levels in the blood were determined at 48 h post- LPS challenge. The data are expressed as the mean ± standard error of the mean (SEM). *n* = 4, 4, 19, or 16 for the PBS+PBS, PBS+TDCA, LPS+PBS, or LPS+TDCA groups, respectively. **(E)** Blood pressure at 4 and 6 h post LPS injection. *n* = 6 but *n* = 5 in PBS+PBS group **(F–H)** The cytokine concentrations in the sera were determined using the cytometric bead array at 6 h post LPS injection or 24 h post CLP. Data shown are pooled from 4 independent experiments for LPS-injection setting or 6 independent experiment for CLP setting with 1~3 mice per experiment. The times (“h”) indicate the interval from the LPS injection or the CLP procedure to time point the data were collected throughout the study. Short horizontal lines among the circles indicate the mean values. *p* < 0.05 using Kaplan-Meier survival analysis and the log-rank test between groups. *p < 0.05 by Student's two-tailed *t*-test. Data shown are pooled from 3 independent experiments with 1~3 mice per experiment otherwise denoted.

When we infused TDCA i.v. (0.5 mg/kg) at 30 min or 24 h after LPS injection, 80 and 50% of the mice survived, respectively (Figure 1A, *p* < 0.05). The TDCA dose of 0.4 mg/kg was sufficient to obtain this effect (Figure S1A). In addition, 70% of the mice survived when we infused TDCA at 2 h after cecal ligation and puncture (CLP, Figure 1B, *p* < 0.05). The plasma Cmax (= 502 ng/ml) of TDCA after i.v. infusion (1 mg/kg) was approximately 1/1,000 of the 50% hemolytic concentration (420 μg/ml) previously reported (32) (Figure S1B) and approximately 1/1,000 less than the cytotoxic dose *in vitro* (33). TDCA did not protect TGR5 KO mice under sepsis (Figure S1C).

TDCA decreased liver and kidney damage in septic mice (Figures 1C,D and Figure S2). H&E staining of the kidney showed that the LPS-injected mice exhibited marked vacuolar degeneration of the tubules (arrows in Figure 1C). TDCA infusion almost completely ameliorated LPS-induced kidney lesions (Figure 1C). The mucopolysaccharides on the basement membranes of the glomerular capillary loops and the tubular epithelium of the kidney were also stained with PAS (arrows in Figure 1C, right column). The loss of the brush border was remarkable in the LPS + PBS group (an arrow) and was significantly recovered by TDCA infusion (an arrow in Figure 1C). TDCA infusion normalized kidney function, liver function and hypotension from 4 h after LPS injection (Figures 1D,E, Figure S2). The production of cytokines, such as TNF-α, MCP-1, IL-6, and IL-1β, was also significantly inhibited by TDCA in both the LPS injection and CLP models (Figures 1F–H, Figure S3).

### The phenotype of CD11B^+^Gr1^hi^ cells increased by TDCA

**Figure 2:**
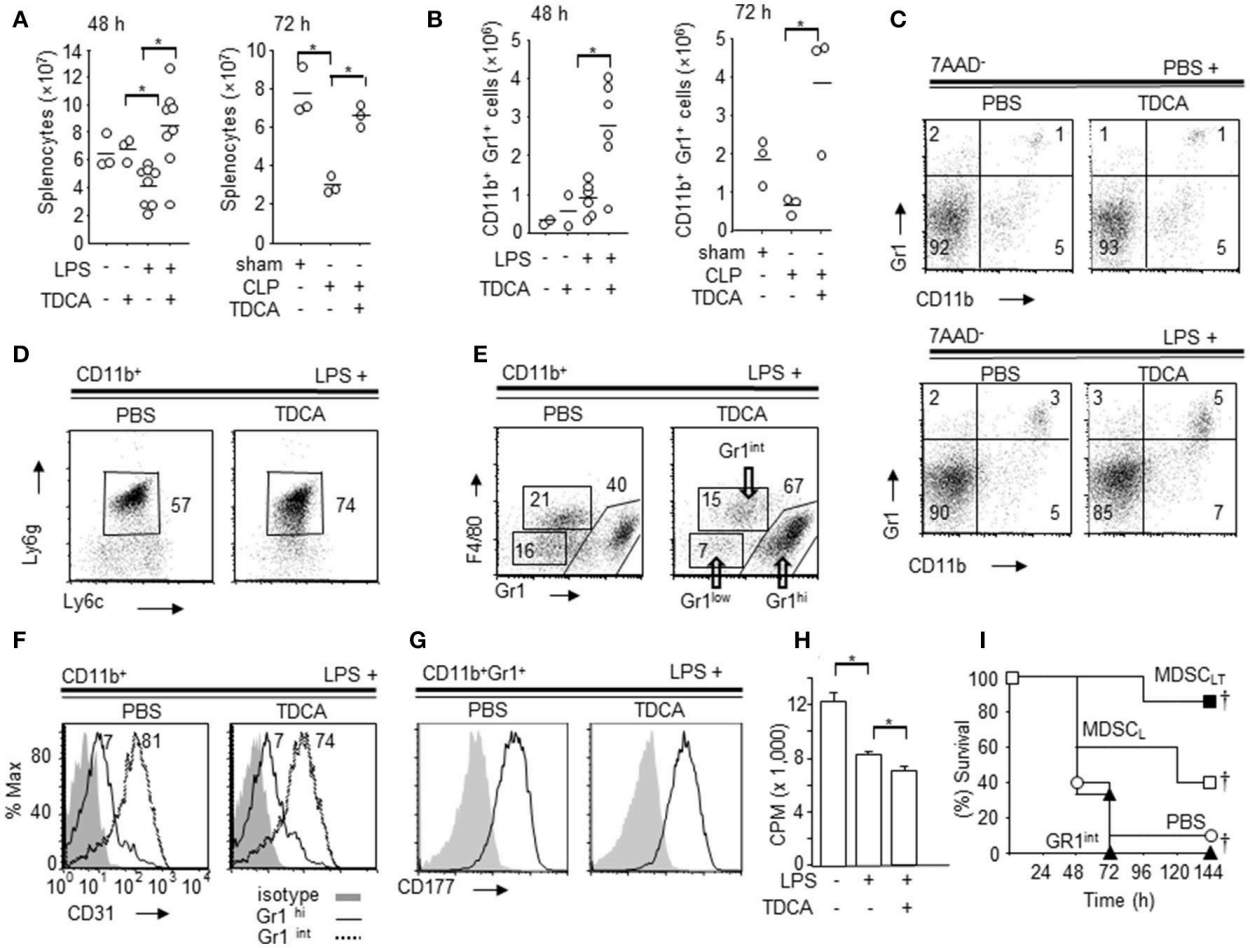
Phenotype of splenic CD11b^+^Gr1^hi^ cells increased by TDCA. **(A)** The absolute numbers of spleen cells from B6 mice at 48 h post LPS injection (left, Data pooled from 5 independent experiments) or 72 h post CLP (right) by Trypan blue exclusion assay. **(B)** The absolute number of splenic CD11b^+^Gr1^hi^ cells from B6 mice at 48 h post LPS injection (left) or 72 h post CLP setting (right). **(C)** Representative FACS plots of **(B)**. **(D)** Representative FACS plots showing the expression of Ly6g and Ly6c on the subpopulation of CD11b^+^ cells. The percentage denotes the % of Ly6c^int^Ly6g^+^ cells among CD11b^+^ cells. **(E)** Representative FACS plots showing the expression of Gr1 and F4/80 on CD11b^+^ cells. **(F)** Representative FACS plots showing expression of CD31 on CD11b^+^Gr1^hi^ cells (solid line) and CD11b^+^Gr1^int^ cells (dotted line) from the LPS+PBS group and LPS+TDCA group. Filled histograms denote the isotype control. **(G)** Expression of CD177 on CD11b^+^Gr1^hi^ cells. Filled histograms denote the isotype control. **(H)** [H^3^]Thymidine-incorporation of T-cells following CD3 + CD28 ligation was measured after co-culture with CD11b^+^Gr1^hi^ cells (E:T = 1:5) purified from spleen of mice injected with PBS + PBS, LPS + PBS, or LPS + TDCA. The data are expressed as the mean count per minute (cpm) ± SEM of triplicate assays pooled from 3 independent experiments. **(I)** The survival of mice after adoptive transfer of CD11b^+^Gr1^hi^ cells (1 × 10^5^ cells/mouse) purified from the LPS + TDCA group (■, *n* = 7, MDSC_LT_) or the LPS + PBS group (□, *n* = 5, MDSC_L_). CD11b^+^Gr1^int^ cells (▴, *n* = 3) were purified from mice in the LPS + TDCA group. The thin horizontal lines on the top of the FACS plots indicate the gates used in the FACS analysis, and the thick horizontal lines are the experimental groups treated with LPS or PBS. The gating strategies are shown in Figures S7~S12. Data shown are pooled from 3 independent experiments with 1~3 mice per experiment otherwise denoted. Representative FACS plots of 3 independent experiments. ^†^*p* < 0.05 using Kaplan-Meier survival analysis and the log-rank test between groups. *p < 0.05 by Student's two-tailed *t*-test.

As previously reported (34), mice with sepsis exhibited reduced splenocyte numbers at 48 h after LPS injection and 72 h after CLP (Figure 2A, Figure S4). However, the total numbers of splenocytes were significantly increased in the LPS + TDCA group at 48 h after LPS injection and 72 h in the CLP + TDCA group. CD11b^+^Gr1^+^ cells increased both in the LPS + TDCA group and CLP + TDCA group (Figures 2B,C, Figures S4, S5). TDCA treatment did not increase the number of T cells or CD11c^+^ cells (Figure S6). There were no significant changes in the number of CD4^+^FoxP3^+^ T_reg_ cells or the expression of CTLA4 on these cells (Figure S7).

In both the LPS injection and CLP settings, CD11b^+^Ly6g^+^Ly6c^int^ cells exhibited a more profound increase in the TDCA group than the PBS group (Figure 2D, Figures S8, S9). In addition, TDCA infusion further increased the % CD11b^+^Gr1^hi^F4/80^int^ cells in LPS injection settings compared with PBS group (Figure 2E, Figure S10). Interestingly, CD31, a marker of immature myeloid progenitors, was expressed on CD11b^+^Gr1^int^ but not on CD11b^+^Gr1^hi^ cells (Figure 2F, Figure S11). The CD11b^+^Gr1^hi^ cells also expressed the neutrophil marker CD177 (Figure 2G, Figure S12). There were no significant differences in the expression of CD31 or CD177 between CD11b^+^Gr1^hi^ cells from the “LPS + TDCA” group (“MDSC_LT_” onwards) and CD11b^+^Gr1^hi^ cells from the “LPS + PBS” group (“MDSC_L_” onwards).

FACS-purified MDSC_LT_ inhibited T cell proliferation following CD3 + CD28 ligation *in vitro* better than MDSC_L_ cells (Figure 2H, *p* < 0.05). Adoptive transfer of FACS-sorted MDSC_LT_ (1 × 10^5^ cells) significantly improved the survival rate compared with MDSC_L_ (Figure 2I, Figure S13, *p* ≤ 0.05). CD11b^+^Gr1^−^ cells (DATA not shown), CD11b^+^Gr1^int^ cells from the LPS + TDCA group or PBS did not confer protection.

### Proteogenomic profiling of MDSC_LT_

**Figure 3:**
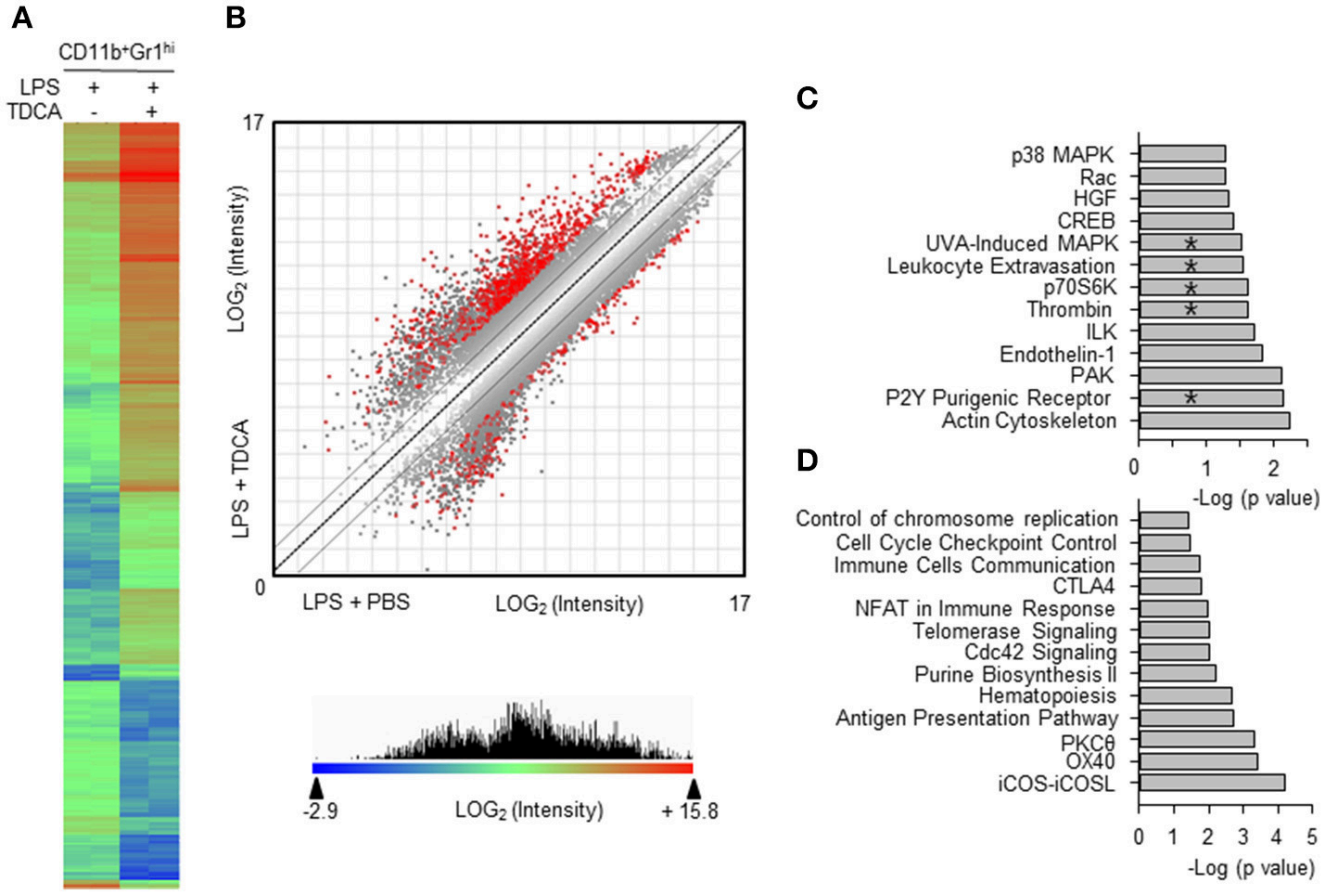
Comparison of gene expression profiles of MDSC_L_ and MDSC_LT_. **(A)** CD11b^+^Gr1^hi^ cells were FACS-purified from the LPS + PBS group (8 independent sorting, 4~9 mice samples were pooled for each sorting) and the LPS + TDCA group (5 independent sorting, 4~6 mice samples were pooled for each sorting). cDNAs from each mouse were pooled and gene expression was profiled in duplicate. Eight hundred eighteen genes were filtered (>8-fold change, *p* < 0.05) from 14,074 genes (*p* < 0.05) and analyzed to generate a heat map and scatter plot. The gene with the lowest expression value is displayed as a highly suppressed gene in blue (signal intensity = 2^−2.9^). The highest expression value is displayed as a highly expressed gene in red (signal intensity = 2^15.8^). The range of expression intensity is color coded and is depicted in the right. **(B)** The double-log scatter plot indicates the signal intensities of all oligo probes. The 818 genes shown in red showed > 8-fold change and *p-*value < 0.05. The best-fit linear trend (dotted line) and the ±2-fold change lines are indicated. **(C)** Four hundred ninety-six genes that exhibited 5-fold down regulation (*p* < 0.05) were extracted, and their pathway associations were investigated. **(D)** Five hundred forty-four genes that exhibited 8-fold up regulation (*p* < 0.05) were extracted, and their pathway associations were investigated using IPA®. The pathways overlapping with the proteomic analysis are denoted by “*”. −Log (*p*-value) was calculated using the right-tailed Fisher's exact test to measure the likelihood that the association between a set of genes and pathway was due to random chance. Pathways with *p* < 0.05 are shown.

The gene expression profiles were compared between MDSC_LT_ and MDSC_L_ (Figure 3A). The microarray results indicated that 818 genes showed more than 8-fold changes in the 95% confidence interval (*p* < 0.05, Figure 3B). These genes were further analyzed to decipher the association with signaling pathways using Ingenuity Pathway Analysis (IPA®, QIAGEN, Redwood City, CA). The expressions of genes for the signaling pathways necessary for immune regulation were controlled by TDCA (*p* < 0.05, Figures 3C,D). In particular, the genes for pro-inflammatory signaling pathways, such as chemotaxis [denoted as Rac signaling (35), leukocyte extravasation, ILK signaling (36), and PAK signaling (37)], MAPK signaling, CREB signaling (38), p70S6K signaling (39), thrombin signaling (40), and P2Y purinergic receptor signaling (41), were significantly down-regulated (Figure 3C). The genes for the antigen presentation pathway and cell cycle control were highly up-regulated (Figure 3D).

**Figure 4:**
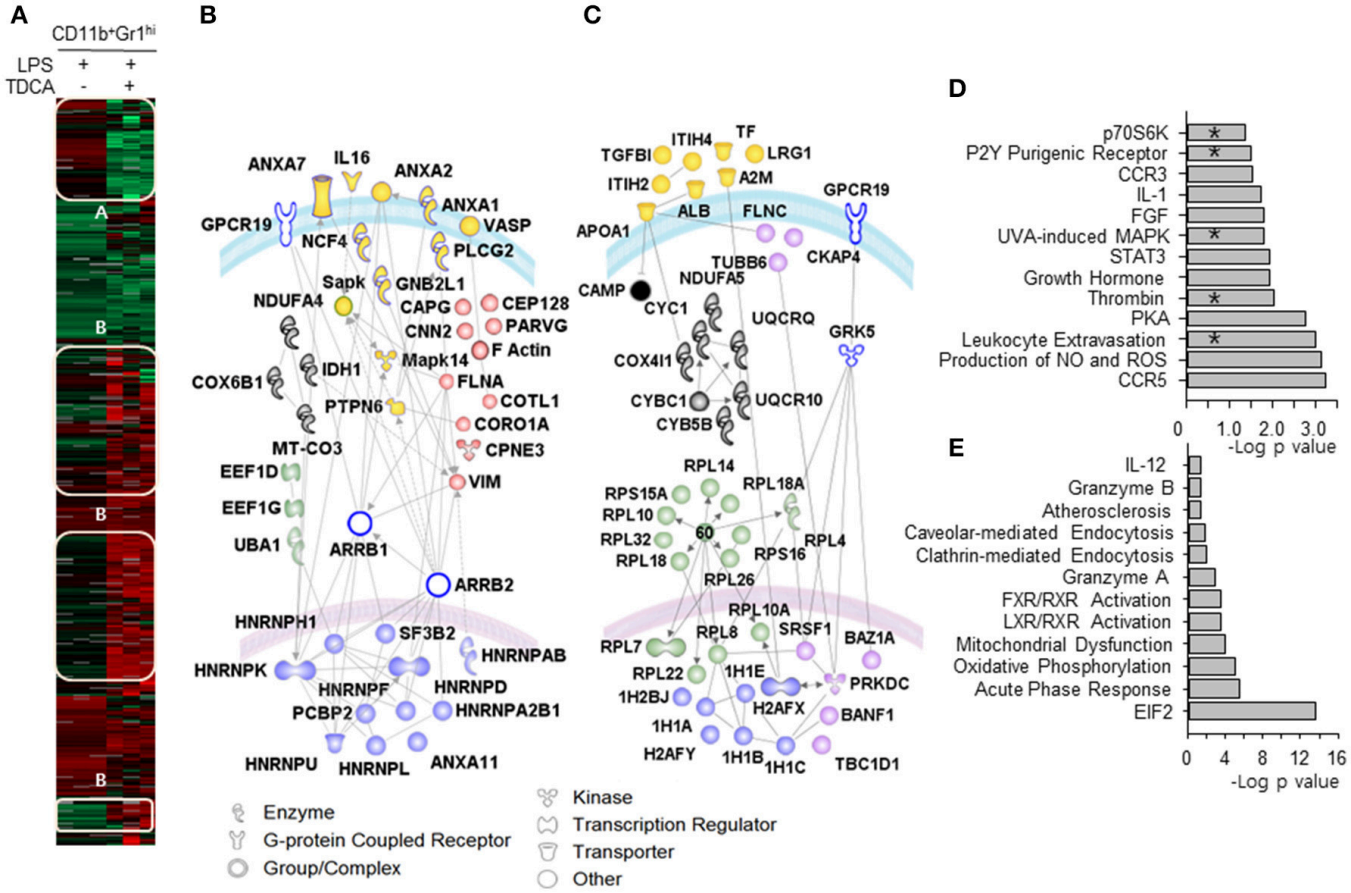
Proteomic differences between MDSC_LT_ and MDSC_L_ cells. **(A)** The heat map indicates two distinct proteome clusters that were down-regulated in MDSC_LT_ (cluster A) and up-regulated in MDSC_LT_ (cluster B) compared with MDSC_L_ cells. Protein lysates were pooled from cells obtained by 3 independent sorting for each group. *n* = 3 mice in each independent sorting. **(B)** The proteins in cluster A were further sub-clustered based on their common functions. The red symbols represent molecules that play roles in cellular trafficking. Blue symbols (alternative splicing), green symbols (protein metabolism), gray symbols (mitochondrial oxidative phosphorylation) and yellow symbols (pro-inflammatory signaling/adhesion/ cytokine molecules) were used to denote each sub-cluster. **(C)** The proteins in cluster B were further sub-clustered as **(B)**. The pink symbols in the nucleus represent molecules that are potentially associated with the cell cycle and differentiation. The blue symbols in the nucleus represent histones for chromatin remodeling. The green symbols (ribosomal proteins), cytosolic pink symbols (cytoskeletal proteins), gray symbols (mitochondrial respiratory chain) and yellow symbols (acute phase response proteins) were color-coded based on their functions listed in the database of Ingenuity Pathway Analysis (IPA®, QIAGEN). Open symbols are molecules predicted by IPA® that were not observed in the experiments. The gray lines represent potential interactions between the molecules predicted by IPA®. The curved blue lines and pink lines represent the cytoplasmic and nuclear membranes, respectively. Canonical signaling pathways for up-regulated proteins **(D)** and down-regulated proteins **(E)** were analyzed. *denotes signaling pathways overlapped with the microarray analysis. –Log (*p*-value) was calculated using the right-tailed Fisher's exact test to measure the likelihood that the association between a set of genes and pathway was due to random chance. Pathways with *p* < 0.05 are shown.

We further compared the proteomic profiles between MDSC_LT_ and MDSC_L_ cells (Figure 4). Using iTRAQ labeling, 1,643 unique proteins were identified at a protein threshold with a 1.0% false discovery rate. Among these proteins, 887 showing a peptide spectral count in more than 2 assays from triplicate assays are depicted in the heat map (Figure 4A). The heat map shows two distinct proteome clusters that were down-regulated in MDSC_LT_ (cluster A) and up-regulated in MDSC_LT_ (cluster B) compared with MDSC_L_ cells. Various heterogeneous ribonucleoproteins (hnRNPs) responsible for the alternative splicing of pre-mRNA were down-regulated (Figure 4B, nuclear blue symbols) (42). The proteins for cytoskeletal rearrangement were down-regulated (Figure 4B, red symbols). A set of histones (1H1C, 1H1E, H2AFX) that repress transcription by preventing transcription factor access (43) were up-regulated in MDSC_LT_ compared with MDSC_L_ cells (Figure 4C, nuclear blue symbols). TDCA treatment resulted in heterogeneous ribosome composition by up-regulating the expression of a set of ribosomal proteins in MDSC_LT_ (Figure 4C, green symbols). Specialized sets of ribosomes with different functions may be complexed because of the differential expression of ribosomal subunit proteins following TDCA treatment (44). The expression of proteome for mitochondrial oxidative phosphorylation was up-regulated (Figure 4C, gray symbols). In addition, CAMP was up-regulated (Figure 4C, cytosolic black symbol), which participates in inhibiting the expression of various pro-inflammatory molecules, such as TNF-α, CXCL8, IL6, CCL2, IL-1β, leukotriene B4 and nitric oxide, by regulating Erk1/2, P38 MAPK, Jnk, Akt, and NF-κB (45). In the proteomic analysis, proteins for chemotaxis and pro-inflammatory pathways were down-regulated, as indicated by microarray analysis (Figure 4D). Proteomic profiling also showed that the expression of various proteins responsible for endocytosis and immune-regulation, such as the acute phase response, EIF2 signaling (46), and LXR/RXR/FXR signaling (47), were up-regulated by TDCA treatment (Figure 4E). In both the microarray and iTraq analyses, the pathways for p70S6K signaling, P2Y purinergic receptor signaling, MAPK signaling, thrombin signaling, and leukocyte extravasation signaling were down-regulated in MDSC_LT_ compared with MDSC_L_.

### Mode of immune regulation and proliferation of MDSC_LT_

**Figure 5:**
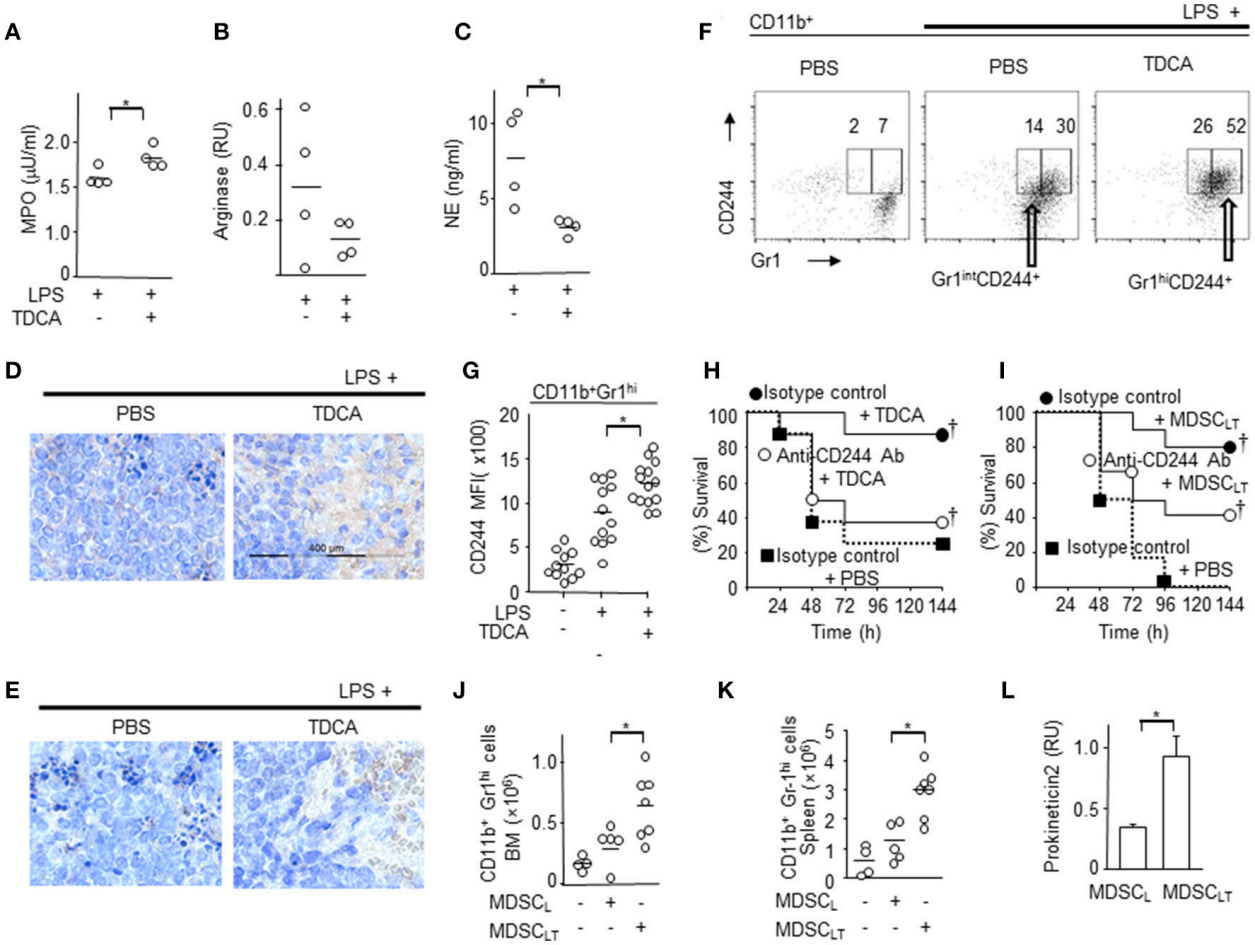
Potential molecular mediators of CD11b^+^Gr1^+^ cells for immune regulatory function. CD11b^+^Gr1^hi^ cells were purified from the spleen and enzyme activity or level of transcripts were determined. **(A)** MPO enzyme activity. **(B)** RT-PCR analysis of arginase gene. **(C)** NE enzyme activity. DATA pooled from 2 independent experiments for A~C. Representative oncostain M **(D)** and lactoferrin **(E)** expression in splenocytes from septic mice determined by immunohistochemistry after treatment with PBS or TDCA. Data shown are representative of 3 independent experiments with 3 samples per experiment. **(F)** Representative FACS plots of **(G)** indicating the surface expression of CD244 on splenic CD11b^+^ Gr1^hi^ cells. The gating strategies are shown in Figure S15. **(G)** MFIs of CD244 expression on CD11b^+^Gr1^hi^ cells are plotted. Data pooled from 4 independent experiments with 2~4 samples per experiment. **(H)** Survival rate of septic mice given LPS i.p. after recieving isotype control antibody + TDCA (•), anti-CD244 antibody + TDCA (°), or isotype control antibody + PBS (■). *n* = 8 **(I)** Survival rate of septic mice that received LPS i.p. after being administered isotype control antibody + adoptive transfer of MDSC_LT_ (•, *n* = 10), anti-CD244 antibody + adoptive transfer of MDSC_LT_ (°, *n* = 12), or isotype control antibody + PBS (■, *n* = 10). The number of CD11b^+^Gr1^hi^ cells in the bone marrow **(J)** or spleen **(K)** after adoptive transfer of MDSC_L_ or MDSC_LT_. The number of total BM cells or total splenocytes were counted and the number of CD11b^+^Gr1^hi^ cells was calculated using the % of cells after FACS analysis as in Figure 2E. **(L)** RT-PCR analysis of prokineticin 2. *n* = 4, RU, relative units of band intensity normalized to the intensity of GAPDH. Short horizontal lines among the circles indicate the mean values. *p < 0.05 by the Student's two-tailed *t*-test. *p* < 0.05 using Kaplan-Meier survival analysis and the log-rank test between groups with the same symbol. Data shown are pooled from 3 independent experiments with 1~4 samples per experiment otherwise denoted.

Based on the proteogenomic profile, we further characterized MDSC_LT_ compared with MDSC_L_. The MPO responsible for ROS generation was higher in MDSC_LT_ than MDSC_L_ cells (Figure 5A). Microarray and RT-PCR analysis of transcripts showed that iNOS levels in both MDSC_LT_ and MDSC_L_ cells were similar to those of normal splenocytes (data not shown). There were no significant differences in the mRNA expression of arginase-1 (*p* = 0.07, Figure 5B). These findings suggest that MDSC_LT_ exerted an immune-regulatory role similar to the role played by gMDSCs using ROS (48). In addition, neutrophil elastase (NE) activity was lower in MDSC_LT_ than MDSC_L_ cells (*p* < 0.05, Figure 5C). Considering that NE plays a role in tissue destruction via extracellular matrix proteolysis, MDSC_LT_ may lead to less tissue destruction than MDSC_L_ cells.

We further characterized the IL-10 production induced by TDCA because IL-10 plays a crucial role in the anti-inflammatory phenotypes of MDSC (49). Interestingly, there was no significant IL-10 production by MDSC_LT_ and MDSC_L_ (Figure S14A). In addition, TDCA treatment conferred protection to IL-10 KO mice comparable to WT mice following LPS challenge (Figure S14B). TDCA treatment after LPS injection did not affect the serum concentration of IL-10 of B6 mice (Figures S14C,D), which suggests that IL-10 may not be involved in the anti-inflammatory effector functions of MDSC_LT_ and TDCA.

The expression levels of oncostatin M (Osm) and lactoferrin, which play crucial roles in innate immune responses, were increased in the spleens of septic mice after TDCA infusion (Figures 5D,E).

Because the microarray data showed increased expression of CD244 in MDSC_LT_ compared with MDSC_L_ (fold change = 2.7 ± 1.1) and the CD244^hi^ MDSC population was significantly increased in tuberculosis patients and in tumor-bearing mice (50, 51), we analyzed the expression of CD244 on MDSC_LT_. FACS analysis also showed that MDSC_LT_ expressed higher levels of CD244 than MDSC_L_ cells (Figures 5F,G and Figure S15). Intravenous injection of anti-CD244 antibody neutralized the protective effect of TDCA infusion *in vivo* (Figure 5H). Because CD244 is expressed on various cell types, we investigated the role of CD244 expressed on MDSC_LT_ after adoptive transfer of MDSC_LT_ into septic mice and i.v. injection of anti-CD244 antibody. Intravenous injection of anti-CD244 antibody abrogated the protective effect of adoptive transfer of MDSC_LT_ cells in the LPS injection setting (Figure 5I).

Adoptive transfer of MDSC_LT_ increased the number of MDSC_LT_ in both the bone marrow (Figure 5J) and spleen (Figure 5K) of recipient B6 mice with sepsis, which suggests that TDCA may induce the production of an autocrine factor necessary for increasing the number of MDSC_LT_ cells. Microarray and RT-PCR analyses showed that MDSC_LT_ expresses prokineticin2 to a greater extent than MDSC_L_ cells, which is crucial for MDSC proliferation (Figure 5L) (52).

## Discussion

Here, we report that TDCA increases the number of immune-regulatory MDSC_LT_ cells, which are distinct from gMDSCs that previously reported, through the global editing of the proteome that increase anti-inflammatory molecules such as MPO, Osm, lactoferrin, and CD244, in addition to prokineticin 2, which are essential for the proliferation of myeloid cells (53). Global editing of the proteome by TDCA decreases pro-inflammatory functions by inhibiting the expression of tissue-degrading enzymes such as neutrophil elastase. In addition, anti-inflammatory molecule expression was increased in MDSC_LT_ compared with MDSC_L_. For example, Osm is involved in anti-inflammatory responses and restores normal homoeostasis after tissue injury or infection (54). Without Osm, LPS induces exaggerated pathogenesis (55). Lactoferrin also possesses anti-oxidant, anti-inflammatory and anti-bacterial functions, which are crucial for protection from sepsis (56). Considering previously published expression profiles (57, 58), immunohistochemistry data showing increased expression of Osm and lactoferrin in spleen might be due to MDSC_LT_ population in spleen (59–63).

Thus, TDCA ameliorates systemic inflammation, normalizes blood pressure, prevents kidney injury and prolongs survival in a mouse sepsis model. Considering that the median plasma concentration of TDCA is 33.9 nM in healthy individuals (64), approximately 10~20 × the physiological plasma levels of TDCA is necessary to inhibit the systemic inflammation incurred by LPS injection or by CLP. The IC50 of TDCA that inhibits the binding of N-^3^H-methylscopolamine to the M3 muscarinic receptor of acetylcholine was 170 μM (33), which suggests that the plasma Cmax after i.v. infusion of 1 mg/kg TDCA is 1/176 × the concentration necessary for antagonizing the M3 muscarinic receptor. The EC50 of deoxycholic acid (DCA) to increase cAMP production in a TGR5-dependent manner was 1 μM (26). This finding suggests that the plasma Cmax of TDCA after 0.5 mg/kg i.v. infusion may be sufficient to activate the TGR5 pathway *in vivo*. Considering together with that TDCA does not protect TGR5 KO mice under sepsis in this study, TDCA might play roles via TGR5 pathway to control sepsis.

TDCA activates the S1PR2 pathway (65) in addition to the TGR5 pathway (24, 26, 30). Activation of the S1PR2 pathway augments pro-inflammatory responses, and TDCA activates the S1PR2 pathway at more than 5 μM (66). Accordingly, it is less likely that S1PR2 plays roles in TDCA-mediated immune regulation, even in various pathological conditions where the plasma concentration of TDCA is less than 1 μM (18).

Conjugated BAs rarely activate nuclear receptors. The most potent activator of FXR, CDCA, had an EC50 of 50 μM in FXR activation, and other conjugated BAs were inactive in this assay at concentrations up to 100 μM (16). Conjugated BAs in fasting blood are typically less than 1 μM with concentrations that reach as high as 5 μM after a meal (4). For these reasons, we hypothesized that the immunomodulatory role of TDCA may be largely dependent on the activation of the TGR5 pathway *in vivo*; however, it needs to be ruled out for interaction with other membrane receptors, such as α1β4/α5β1 integrin, mAChRs and large conductance Ca^2+^-activated K^+^ channels using a panel of loss-of-function or gain-of-function studies in future investigations.

When rats were injected with TDCA i.v. once every day for 28 days, the no-observed-adverse-effect level (NOAEL) was >10 mg/kg (67). Taken together with the toxic dose ranges previously reported (32), the pharmacological effects of TDCA are observed at less than 10 × the plasma concentration of healthy individuals, which is far less than the toxic dose ranges (32).

TDCA-induced CD11b^+^Gr1^hi^CD31^−^CD177^+^ MDSC_LT_ cells are distinctive from the conventional gMDSC subsets present in tumors and inflammatory conditions. In the tumor microenvironment, the CD11b^+^Gr1^hi^CD31^−^ gMDSC subset is less immunosuppressive than the CD11b^+^Gr1^int^CD31^+^ mMDSC subset (68). In comparison, TDCA-induced CD11b^+^Gr1^hi^CD31^−^ MDSC_LT_ cells are more anti-inflammatory than MDSC_L_ cells, which increased under inflammatory conditions without TDCA (conventional gMDSCs). Furthermore, in terms of anti-inflammatory potency, TDCA-induced CD11b^+^Gr1^int^CD31^+^ cells (with a surface phenotype of conventional mMDSCs) did exhibited reduced anti-inflammatory functions compared with MDSC_LT_ or MDSC_L_ cells. These findings suggest that the phenotype and function of the TDCA-induced MDSC_LT_ cells are different from the gMDSC and mMDSC subsets in tumor-bearing mice and gMDSCs present in inflammatory conditions (68).

The CD244 expression on MDSC_LT_ was substantially higher than that of MDSC_L_. CD244 - CD48 interactions have been reported as both activating and inhibitory, depending on the context or the isoform of CD244 engaged (69). Regardless of the contradictory roles of CD244, several reports suggest critical roles for CD244 - CD48 interactions in immune regulation. CD244 on intraepithelial lymphocytes inhibits inflammatory colitis (70). In addition, the risks of rheumatoid arthritis and systemic lupus erythematosus are increased in patients with genetic polymorphisms in CD244 (71). Moreover, mutation of the CD244 gene is closely linked to the risk of autoimmunity (72).

In this study, we identified an increase in the expression of CD244 on MDSC_LT_ and neutralization of the regulatory activity of MDSC_LT_ by anti-CD244 antibody treatment. Thus, CD244 on MDSC_LT_ may play a crucial role in inhibiting the function of other inflammatory cells as an immune checkpoint. However, detailed loss-of-function studies are necessary to assess the role of CD244 expressed on MDSC_LT_ in sepsis because CD244 also plays crucial roles in NK cell functions and NK cells contribute to antibacterial immunity via crosstalk with other immune cells (73). For these reasons, the homotypic interaction of CD244 and heterotypic CD244-CD48 interactions between various hematopoietic cells must be examined to elucidate protective roles of TDCA.

**Figure 6:**
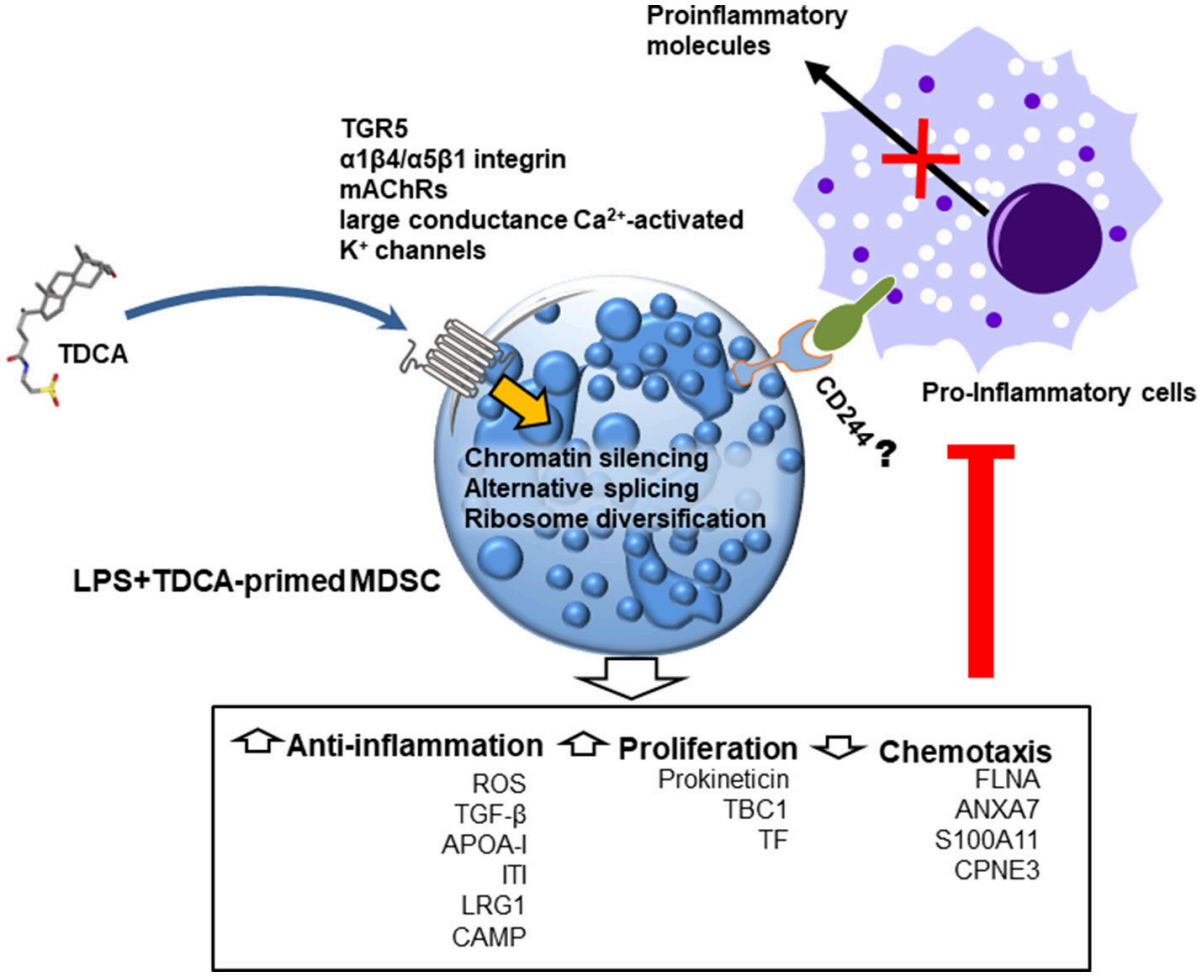
Schematic diagram indicating the potential anti-inflammatory mode of action of TDCA in mice with sepsis. When administered, TDCA edits the transcription/translation machinery and reprograms CD11b^+^Gr1^hi^ cells to regulate inflammation and proliferation. In future studies, the respective roles of known TDCA receptors (such as TGR5, α1β4/α5β1 integrin, mAChRs and large conductance Ca^2+^-activated K^+^ channels) must be tested in immuno-regulation in response to TDCA.

Proteogenomic analysis suggests three potential transcriptional and translational control mechanisms for global proteome editing by TDCA (Figure 6). First, increases in linker histones by TDCA may inhibit the binding of various transcription factors to chromosomes (43). Second, the expression of various hnRNPs diversified by TDCA may change of the mRNA repertoire by reprogramming alternative splicing (42). Finally, the heterogeneous ribosome composition incurred by TDCA generates new sets of functional proteomes (44). In this manner, global editing of the proteome may be responsible for the down-regulation of pro-inflammatory molecules that play roles in chemotaxis, thrombin signaling (74), IL-1 signaling and purinergic responses (75) and the up-regulation of anti-inflammatory responses (notably APR, ROS generation and FXR), as well as increased hematopoiesis.

One question remains elusive. How does TDCA inhibit inflammation and yet control bacteria in mice CLP model that are infected with live bacteria? Because inhibition in a pro-inflammatory response leaves that animal at a significant disadvantage and should lead to increased sensitivity to infection. Our proteomics and immunohistochemistry data provides plausible bacterial clearance mechanisms exerted by TDCA. TDCA increases expression of MPO, lactoferrin and oncostatin M (Figure 5). These molecules play roles in bacterial clearance (59–63). MPO is essential in oxidative killing of bacteria in phagolysosomes (76). MPO generates lethal anti-microbial oxidants that react with ingested bacteria to kill them (76). Lactoferrin is a non-haem iron-binding protein (77). Thus, lactoferrin sequestrate iron in sites of infection, which deprives the microorganism of iron essential for living, thus killing bacteria (78). Lactoferrin also interact with the cellular membrane of infectious agent directly and cause bacterial lysis (78). Oncostatin M is responsible for increased levels of liver iron regulatory hormone hepcidin and decreases serum iron levels that hampers bacterial growth (79). Although many reports showed bacterial clearance by these molecules, we need to decipher exact mechanism of bacterial clearance by these molecules when we infused TDCA into mice under sepsis.

More than 2 million individuals worldwide suffer from sepsis on an annual basis (80). Because a plethora of pathogenic signaling pathways are simultaneously activated in septic patients, clinical trials targeting a single inflammatory mediator, coagulation factor or pro-inflammatory signal transducer have not shown significant survival benefits (81). In contrast to former strategies examining the blockade of pro-inflammatory pathways, targeting intrinsic immune regulatory mechanisms may be more effective for inhibiting the broad spectrum of pathways that are activated in sepsis (82). For these reasons, *in vivo* expansion of MDSC_LT_ using a pharmacological dose of TDCA may be a plausible approach to inhibit the broad-spectrum pathogenesis exhibited in septic patients.

## Author contributions

SC contributed to the design and analysis of the FACS experiments in addition to adoptive transfer experiments. Y-HK investigated the role of TDCA in terms of blood pressure, cytokine production, and survival using the LPS injection and CLP models, performed the animal work in the adoptive transfer experiments and determined the surface phenotypes of immune cells by FACS. Y-JK determined the intracellular phenotype of immune cells by FACS and performed the splenocyte preparation and FACS staining for FACS sorting in the adoptive transfer experiments. Y-WK studied effects of TDCA using TGR5 KO mice. SM, YL, JJ, DH, and YK contributed to the proteomic analysis. H-EJ was involved in the gene expression studies using microarray and RT-PCR. J-AC and T-JK analyzed the protein expression using western blotting. T-CC and N-HC studied the tissues using confocal microscopy. H-JM supported unspecified basic laboratory work. H-RK assisted with the FACS sorting. YN and S-HS studied the tissues after staining with H&E and PAS. H-CL and E-HN were involved in the gene expression studies. HC and MC analyzed the microarray data. NM provided guidance for the neutrophil studies and the antibodies necessary for neutrophil staining. All authors contributed to the manuscript preparation. S-YS supervised the study, analyzed the data and wrote the manuscript.

### Conflict of interest statement

The patent “Use of biological surfactant as anti-inflammatory agent and tissue preservative solution: US 20100267684 A1” was invented by Seong et al. and applied by Seoul National University R&DB Foundation. The exclusive license for the patent was transferred from SNU R&DB to Shaperon Inc. SS is a founder of Shaperon Inc. and CEO currently. The remaining authors declare that the research was conducted in the absence of any commercial or financial relationships that could be construed as a potential conflict of interest.

## References

[1] Russell DW. The enzymes, regulation, and genetics of bile acid synthesis. Annu Rev Biochem. (2003) 72:137–74. 10.1146/annurev.biochem.72.121801.161712 12543708

[2] Lieu T Jayaweera G Bunnett NW. GPBA: a GPCR for bile acids and an emerging therapeutic target for disorders of digestion and sensation. Br J Pharmacol. (2014) 171:1156–66. 10.1111/bph.12426 24111923 PMC3952795

[3] Schaap FG Trauner M Jansen PL. Bile acid receptors as targets for drug development. Nat Rev Gastroenterol Hepatol. (2014) 11:55–67. 10.1038/nrgastro.2013.151 23982684

[4] Copple BL Li T. Pharmacology of bile acid receptors: evolution of bile acids from simple detergents to complex signaling molecules. Pharmacol Res. (2016) 104:9–21. 10.1016/j.phrs.2015.12.007 26706784 PMC4900180

[5] Dopico AM Walsh JV Jr Singer JJ. Natural bile acids and synthetic analogues modulate large conductance Ca^2+^-activated K^+^ (BKCa) channel activity in smooth muscle cells. J Gen Physiol. (2002) 119:251–73. 10.1085/jgp.20028537 11865021 PMC2217287

[6] Raufman JP Chen Y Cheng K Compadre C Compadre L Zimniak P. Selective interaction of bile acids with muscarinic receptors: a case of molecular mimicry. Eur J Pharmacol. (2002) 457:77–84. 10.1016/S0014-2999(02)02690-0 12464352

[7] Shaik FB Prasad DV Narala VR. Role of farnesoid X receptor in inflammation and resolution. Inflamm Res. (2015) 64:9–20. 10.1007/s00011-014-0780-y 25376338

[8] Greve JW Gouma DJ Buurman WA. Bile acids inhibit endotoxin-induced release of tumor necrosis factor by monocytes: an *in vitro* study. Hepatology (1989) 10:454–8. 10.1002/hep.1840100409 2777206

[9] Ferrari C Macchiarulo A Costantino G Pellicciari R. Pharmacophore model for bile acids recognition by the FPR receptor. J Comput Aided Mol Des. (2006) 20:295–303. 10.1007/s10822-006-9055-1 16972170

[10] Guo C Xie S Chi Z Zhang J Liu Y Zhang L Bile Acids Control Inflammation and Metabolic Disorder through Inhibition of NLRP3 Inflammasome. Immunity (2016) 45:802–16. 10.1016/j.immuni.2016.09.008 27692610

[11] Kwong E Li Y Hylemon PB Zhou H. Bile acids and sphingosine-1-phosphate receptor 2 in hepatic lipid metabolism. Acta Pharm Sin B (2015) 5:151–7. 10.1016/j.apsb.2014.12.009 26579441 PMC4629213

[12] Parks DJ Blanchard SG Bledsoe RK Chandra G Consler TG Kliewer SA. Bile acids: natural ligands for an orphan nuclear receptor. Science (1999) 284:1365–8. 10.1126/science.284.5418.1365 10334993

[13] Staudinger JL Goodwin B Jones SA Hawkins-Brown D MacKenzie KI LaTour A. The nuclear receptor PXR is a lithocholic acid sensor that protects against liver toxicity. Proc Natl Acad Sci USA. (2001) 98:3369–74. 10.1073/pnas.051551698 11248085 PMC30660

[14] Xie W Radominska-Pandya A Shi Y Simon CM Nelson MC Ong ES. An essential role for nuclear receptors SXR/PXR in detoxification of cholestatic bile acids. Proc Natl Acad Sci USA. (2001) 98:3375–80. 10.1073/pnas.051014398 11248086 PMC30661

[15] Makishima M Lu TT Xie W Whitfield GK Domoto H Evans RM. Vitamin D receptor as an intestinal bile acid sensor. Science (2002) 296:1313–6. 10.1126/science.1070477 12016314

[16] Makishima M Okamoto AY Repa JJ Tu H Learned RM Luk A. Identification of a nuclear receptor for bile acids. Science (1999) 284:1362–5. 10.1126/science.284.5418.1362 10334992

[17] Wang YD Chen WD Huang W. FXR, a target for different diseases. Histol Histopathol. (2008) 23:621–7. 10.14670/HH-23.621 18283647

[18] Sugita T Amano K Nakano M Masubuchi N Sugihara M Matsuura T. Analysis of the serum bile acid composition for differential diagnosis in patients with liver disease. Gastroenterol Res Pract. (2015) 2015:717431. 10.1155/2015/717431 25821461 PMC4363704

[19] Perino A Schoonjans K. TGR5 and Immunometabolism: insights from physiology and pharmacology. Trends Pharmacol Sci. (2015) 36:847–57. 10.1016/j.tips.2015.08.002 26541439

[20] Guo C Qi H Yu Y Zhang Q Su J Yu D. The G-protein-coupled bile acid receptor Gpbar1 (TGR5) inhibits gastric inflammation through antagonizing NF-kappaB signaling pathway. Front Pharmacol. (2015) 6:287. 10.3389/fphar.2015.00287 26696888 PMC4675858

[21] McMillin M Frampton G Tobin R Dusio G Smith J Shin H. TGR5 signaling reduces neuroinflammation during hepatic encephalopathy. J Neurochem. (2015) 135:565–76. 10.1111/jnc.13243 26179031 PMC5031412

[22] Wang YD Chen WD Yu D Forman BM Huang W. The G-Protein-coupled bile acid receptor, Gpbar1 (TGR5), negatively regulates hepatic inflammatory response through antagonizing nuclear factor kappa light-chain enhancer of activated B cells (NF-kappaB) in mice. Hepatology (2011) 54:1421–32. 10.1002/hep.24525 21735468 PMC3184183

[23] Hov JR Keitel V Laerdahl JK Spomer L Ellinghaus E ElSharawy A. Mutational characterization of the bile acid receptor TGR5 in primary sclerosing cholangitis. PLoS ONE (2010) 5:e12403. 10.1371/journal.pone.0012403 20811628 PMC2928275

[24] Pols TW Noriega LG Nomura M Auwerx J Schoonjans K. The bile acid membrane receptor TGR5 as an emerging target in metabolism and inflammation. J Hepatol. (2011) 54:1263–72. 10.1016/j.jhep.2010.12.004 21145931 PMC3650458

[25] Guo C Chen WD Wang YD TGR5, Not only a metabolic regulator. Front Physiol. (2016) 7:646 10.3389/fphys.2016.00646 28082913 PMC5183627

[26] Kawamata Y Fujii R Hosoya M Harada M Yoshida H Miwa M. A G protein-coupled receptor responsive to bile acids. J Biol Chem. (2003) 278:9435–40. 10.1074/jbc.M209706200 12524422

[27] Zhong M. TGR5 as a therapeutic target for treating obesity. Curr Top Med Chem. (2010) 10:386–96. 10.2174/156802610790980576 20180762

[28] Attili AF Angelico M Cantafora A Alvaro D Capocaccia L. Bile acid-induced liver toxicity: relation to the hydrophobic-hydrophilic balance of bile acids. Med Hypotheses (1986) 19:57–69. 10.1016/0306-9877(86)90137-4 2871479

[29] Lamireau T Zoltowska M Levy E Yousef I Rosenbaum J Tuchweber B. Effects of bile acids on biliary epithelial cells: proliferation, cytotoxicity, and cytokine secretion. Life Sci. (2003) 72:1401–11. 10.1016/S0024-3205(02)02408-6 12527037

[30] Watanabe M Houten SM Mataki C Christoffolete MA Kim BW Sato H. Bile acids induce energy expenditure by promoting intracellular thyroid hormone activation. Nature (2006) 439:484–9. 10.1038/nature04330 16400329

[31] Kersey PJ Duarte J Williams A Karavidopoulou Y Birney E Apweiler R. The international protein index: an integrated database for proteomics experiments. Proteomics (2004) 4:1985–8. 10.1002/pmic.200300721 15221759

[32] Mrowczynska L Bielawski J. The mechanism of bile salt-induced hemolysis. Cell Mol Biol Lett. (2001) 6:881–95. 11753435

[33] Raufman JP Chen Y Zimniak P Cheng K. Deoxycholic acid conjugates are muscarinic cholinergic receptor antagonists. Pharmacology (2002) 65:215–21. 10.1159/000064347 12119452

[34] Hotchkiss RS Tinsley KW Swanson PE Schmieg RE Jr Hui JJ Chang KC. Sepsis-induced apoptosis causes progressive profound depletion of B and CD4^+^ T lymphocytes in humans. J Immunol. (2001) 166:6952–63. 10.4049/jimmunol.166.11.6952 11359857

[35] Steffen A Ladwein M Dimchev GA Hein A Schwenkmezger L Arens S Rac function is crucial for cell migration but is not required for spreading and focal adhesion formation. J Cell Sci. (2013) 126:4572–88. 10.1242/jcs.118232 23902686 PMC3817791

[36] Moik D Bottcher A Makhina T Grashoff C Bulus N Zent R. Mutations in the paxillin-binding site of integrin-linked kinase (ILK) destabilize the pseudokinase domain and cause embryonic lethality in mice. J Biol Chem. (2013) 288:18863–71. 10.1074/jbc.M113.470476 23658024 PMC3696662

[37] Byrne KM Monsefi N Dawson JC Degasperi A Bukowski-Wills JC Volinsky N. Bistability in the Rac1, PAK, and RhoA signaling network drives actin cytoskeleton dynamics and cell motility switches. Cell Syst. (2016) 2:38–48. 10.1016/j.cels.2016.01.003 27136688 PMC4802415

[38] Newton K Dixit VM. Signaling in innate immunity and inflammation. Cold Spring Harb Perspect Biol. (2012) 4:a006049. 10.1101/cshperspect.a006049 22296764 PMC3282411

[39] Qin X Jiang X Jiang X Wang Y Miao Z He W. Micheliolide inhibits LPS-induced inflammatory response and protects mice from LPS challenge. Sci Rep. (2016) 6:23240. 10.1038/srep23240 26984741 PMC4794649

[40] Foley JH Conway EM. Cross Talk pathways between coagulation and inflammation. Circ Res. (2016) 118:1392–408. 10.1161/CIRCRESAHA.116.306853 27126649

[41] Cekic C Linden J. Purinergic regulation of the immune system. Nat Rev Immunol. (2016) 16:177–92. 10.1038/nri.2016.4 26922909

[42] Lynch KW. Consequences of regulated pre-mRNA splicing in the immune system. Nat Rev Immunol. (2004) 4:931–40. 10.1038/nri1497 15573128

[43] Croston GE Kerrigan LA Lira LM Marshak DR Kadonaga JT. Sequence-specific antirepression of histone H1-mediated inhibition of basal RNA polymerase II transcription. Science (1991) 251:643–9. 10.1126/science.1899487 1899487

[44] Xue S Barna M. Specialized ribosomes: a new frontier in gene regulation and organismal biology. Nat Rev Mol Cell Biol. (2012) 13:355–69. 10.1038/nrm3359 22617470 PMC4039366

[45] Ishida W Harada Y Fukuda K Fukushima A. Inhibition by the antimicrobial peptide LL37 of lipopolysaccharide-induced innate immune responses in human corneal fibroblasts. Invest Ophthalmol Vis Sci. (2016) 57:30–9. 10.1167/iovs.15-17652 26746016

[46] Carpenter S Ricci EP Mercier BC Moore MJ Fitzgerald KA. Post-transcriptional regulation of gene expression in innate immunity. Nat Rev Immunol. (2014) 14:361–76. 10.1038/nri3682 24854588

[47] Zhu C Fuchs CD Halilbasic E Trauner M Bile acids in regulation of inflammation and immunity: friend or foe? Clin Exp Rheumatol. (2016) 34:25–31. 27586800

[48] Pillay J Tak T Kamp VM Koenderman L. Immune suppression by neutrophils and granulocytic myeloid-derived suppressor cells: similarities and differences. Cell Mol Life Sci. (2013) 70:3813–27. 10.1007/s00018-013-1286-4 23423530 PMC3781313

[49] Brudecki L Ferguson DA McCall CE El Gazzar M Myeloid-derived suppressor cells evolve during sepsis and can enhance or attenuate the systemic inflammatory response. Infect Immun. (2012) 80:2026–34. 10.1128/IAI.00239-12 22451518 PMC3370575

[50] Yang B Wang X Jiang J Zhai F Cheng X. Identification of CD244-expressing myeloid-derived suppressor cells in patients with active tuberculosis. Immunol Lett. (2014) 158:66–72. 10.1016/j.imlet.2013.12.003 24333340

[51] Youn JI Collazo M Shalova IN Biswas SK Gabrilovich DI. Characterization of the nature of granulocytic myeloid-derived suppressor cells in tumor-bearing mice. J Leukoc Biol. (2012) 91:167–81. 10.1189/jlb.0311177 21954284 PMC3250305

[52] Gabrilovich DI Nagaraj S. Myeloid-derived suppressor cells as regulators of the immune system. Nat Rev Immunol. (2009) 9:162–74. 10.1038/nri2506 19197294 PMC2828349

[53] Xin H Lu R Lee H Zhang W Zhang C Deng J. G-protein-coupled receptor agonist BV8/prokineticin-2 and STAT3 protein form a feed-forward loop in both normal and malignant myeloid cells. J Biol Chem. (2013) 288:13842–9. 10.1074/jbc.M113.450049 23548897 PMC3650420

[54] Tanaka M Miyajima A. Oncostatin M, a multifunctional cytokine. Rev Physiol Biochem Pharmacol. (2003) 149:39–52. 10.1007/s10254-003-0013-1 12811586

[55] Glezer I Rivest S. Oncostatin M is a novel glucocorticoid-dependent neuroinflammatory factor that enhances oligodendrocyte precursor cell activity in demyelinated sites. Brain Behav Immun. (2010) 24:695–704. 10.1016/j.bbi.2010.01.005 20083191

[56] Kruzel ML Actor JK Radak Z Bacsi A Saavedra-Molina A Boldogh I. Lactoferrin decreases LPS-induced mitochondrial dysfunction in cultured cells and in animal endotoxemia model. Innate Immun. (2010) 16:67–79. 10.1177/1753425909105317 19723832 PMC3030479

[57] Rosa L Cutone A Lepanto MS Paesano R Valenti P. Lactoferrin: a natural glycoprotein involved in iron and inflammatory homeostasis. Int J Mol Sci. (2017) 18:E1985. 10.3390/ijms18091985 28914813 PMC5618634

[58] Tamura S Morikawa Y Miyajima A Senba E. Expression of oncostatin M in hematopoietic organs. Dev Dyn. (2002) 225:327–31. 10.1002/dvdy.10156 12412016

[59] Slauch JM. How does the oxidative burst of macrophages kill bacteria? Still an open question. Mol Microbiol. (2011) 80:580–3. 10.1111/j.1365-2958.2011.07612.x 21375590 PMC3109634

[60] Schairer DO Chouake JS Nosanchuk JD Friedman AJ. The potential of nitric oxide releasing therapies as antimicrobial agents. Virulence (2012) 3:271–9. 10.4161/viru.20328 22546899 PMC3442839

[61] Hirche TO Benabid R Deslee G Gangloff S Achilefu S Guenounou M. Neutrophil elastase mediates innate host protection against *Pseudomonas aeruginosa*. J Immunol. (2008) 181:4945–54. 10.4049/jimmunol.181.7.4945 18802098

[62] Zagulski T Jarzabek Z Zagulska A Jaszczak M Kochanowska IE Zimecki M Lactoferrin stimulates killing and clearance of bacteria but does not prevent mortality of diabetic mice. Arch Immunol Ther Exp (Warsz) (2001) 49:431–8. 11814237

[63] Traber KE Hilliard KL Allen E Wasserman GA Yamamoto K Jones MR. Induction of STAT3-Dependent CXCL5 expression and neutrophil recruitment by oncostatin-M during pneumonia. Am J Respir Cell Mol Biol. (2015) 53:479–88. 10.1165/rcmb.2014-0342OC 25692402 PMC4742898

[64] Frommherz L Bub A Hummel E Rist MJ Roth A Watzl B. age-related changes of plasma bile acid concentrations in healthy adults–results from the cross-sectional KarMeN study. PLoS ONE (2016) 11:e0153959. 10.1371/journal.pone.0153959 27092559 PMC4836658

[65] Studer E Zhou X Zhao R Wang Y Takabe K Nagahashi M. Conjugated bile acids activate the sphingosine-1-phosphate receptor 2 in primary rodent hepatocytes. Hepatology (2012) 55:267–76. 10.1002/hep.24681 21932398 PMC3245352

[66] Zhang G Yang L Kim GS Ryan K Lu S O'Donnell RK. Critical role of sphingosine-1-phosphate receptor 2 (S1PR2) in acute vascular inflammation. Blood (2013) 122:443–55. 10.1182/blood-2012-11-467191 23723450 PMC3716205

[67] Choi HJ JW Y Kim YH Kwon E Hyona MK Kim JY Evaluation of acute and subacute toxicity of sodium taurodeoxycholate in rats. Drug Chem Toxicol. (in press). 10.1080/01480545.2019.1609493 31215257

[68] Dolcetti L Peranzoni E Ugel S Marigo I Fernandez Gomez A Mesa C. Hierarchy of immunosuppressive strength among myeloid-derived suppressor cell subsets is determined by GM-CSF. Eur J Immunol. (2010) 40:22–35. 10.1002/eji.200939903 19941314

[69] Lee KM McNerney ME Stepp SE Mathew PA Schatzle JD Bennett M. 2B4 acts as a non-major histocompatibility complex binding inhibitory receptor on mouse natural killer cells. J Exp Med. (2004) 199:1245–54. 10.1084/jem.20031989 15123744 PMC2211902

[70] O'Keeffe MS Song JH Liao G De Calisto J Halibozek PJ Mora JR. SLAMF4 Is a negative regulator of expansion of cytotoxic intraepithelial CD8+ T cells that maintains homeostasis in the small intestine. Gastroenterology (2015) 148:991–1001 e4. 10.1053/j.gastro.2015.02.003 25678452 PMC4409516

[71] Sharp AJ Mefford HC Li K Baker C Skinner C Stevenson RE. A recurrent 15q13.3 microdeletion syndrome associated with mental retardation and seizures. Nature Genetics (2008) 40:322–8. 10.1038/ng.93 18278044 PMC2365467

[72] Kim JR Mathew SO Patel RK Pertusi RM Mathew PA. Altered expression of signalling lymphocyte activation molecule (SLAM) family receptors CS1 (CD319) and 2B4 (CD244) in patients with systemic lupus erythematosus. Clin Exp Immunol. (2010) 160:348–58. 10.1111/j.1365-2249.2010.04116.x 20345977 PMC2883105

[73] Souza-Fonseca-Guimaraes F Adib-Conquy M Cavaillon JM Natural killer (NK) cells in antibacterial innate immunity: angels or devils? Mol Med. (2012) 18:270–85. 10.2119/molmed.2011.00201 22105606 PMC3324953

[74] Ortiz-Stern A Deng X Smoktunowicz N Mercer PF Chambers RC. PAR-1-dependent and PAR-independent pro-inflammatory signaling in human lung fibroblasts exposed to thrombin. J Cell Physiol. (2012) 227:3575–84. 10.1002/jcp.24061 22278285

[75] Jacob F Perez Novo C Bachert C Van Crombruggen K. Purinergic signaling in inflammatory cells: P2 receptor expression, functional effects, and modulation of inflammatory responses. Purinergic Signal (2013) 9:285–306. 10.1007/s11302-013-9357-4 23404828 PMC3757148

[76] Klebanoff SJ Kettle AJ Rosen H Winterbourn CC Nauseef WM. Myeloperoxidase: a front-line defender against phagocytosed microorganisms. J Leukoc Biol. (2013) 93:185–98. 10.1189/jlb.0712349 23066164 PMC3545676

[77] Arslan SY Leung KP Wu CD. The effect of lactoferrin on oral bacterial attachment. Oral Microbiol Immunol. (2009) 24:411–6. 10.1111/j.1399-302X.2009.00537.x 19702956

[78] Gonzalez-Chavez SA Arevalo-Gallegos S Rascon-Cruz Q. Lactoferrin: structure, function and applications. Int J Antimicrob Agents (2009) 33:301.e1–8. 10.1016/j.ijantimicag.2008.07.020 18842395

[79] Chung B Verdier F Matak P Deschemin JC Mayeux P Vaulont S. Oncostatin M is a potent inducer of hepcidin, the iron regulatory hormone. FASEB J. (2010) 24:2093–103. 10.1096/fj.09-152561 20124431

[80] Levy MM Dellinger RP Townsend SR Linde-Zwirble WT Marshall JC Bion J. The surviving sepsis campaign: results of an international guideline-based performance improvement program targeting severe sepsis. Intensive Care Med. (2010) 36:222–31. 10.1007/s00134-009-1738-3 20069275 PMC2826633

[81] Riedemann NC Guo RF Ward PA. Novel strategies for the treatment of sepsis. Nat Med. (2003) 9:517–24. 10.1038/nm0503-517 12724763

[82] Hotchkiss RS Coopersmith CM McDunn JE Ferguson TA. The sepsis seesaw: tilting toward immunosuppression. Nat Med. (2009) 15:496–7. 10.1038/nm0509-496 19424209 PMC3786779

