# Supplementary material for: Taurodeoxycholate Increases the Number of Myeloid-Derived Suppressor Cells That Ameliorate Sepsis in Mice
Source: Front Immunol. 2018 Sep 18;9:1984. doi: 10.3389/fimmu.2018.01984 (PMC6153344; doi:10.3389/fimmu.2018.01984)
Supplement: Supplementary file 1 [file Data_Sheet_1.PDF]

Supplementary Material

Supplementary FIGURES

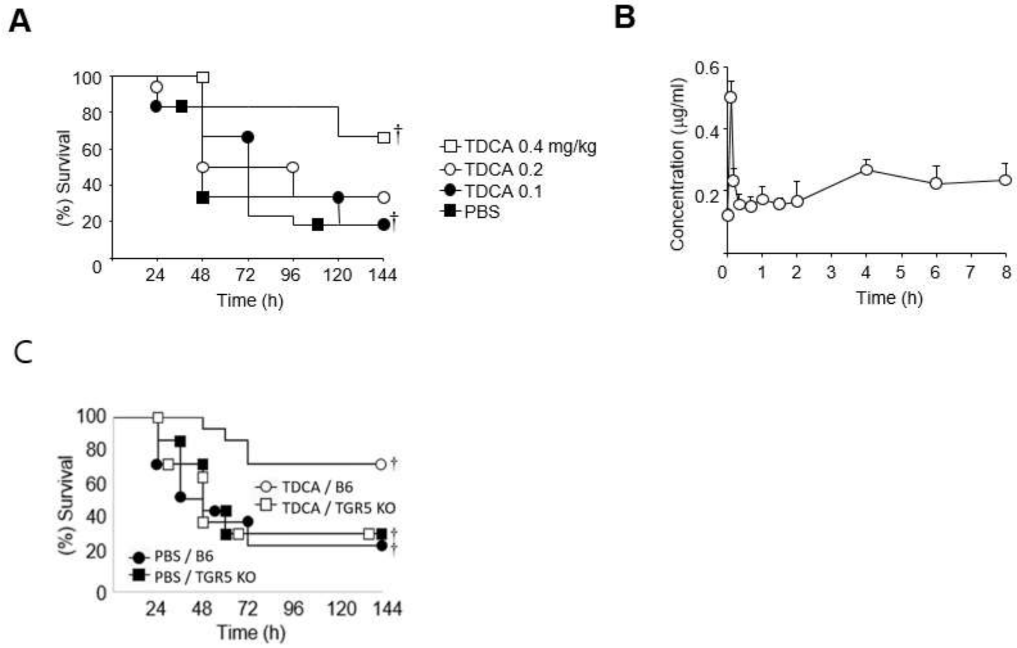

Figure S1. Survival of mice following the infusion of variable doses of TDCA i.v. and the plasma concentration of TDCA. (A) Dose-survival curve of mice undergoing sepsis treated with TDCA (n=6/group for TDCA and n=5 for PBS). Data shown are pooled from 2 independent experiments with 2~3 mice per experiment. (B) Plasma concentration of TDCA (n=3) after i.v. infusion of 1 mg/kg TDCA. † indicates p<0.05 compared by Kaplan-Meier survival analysis and the log-rank test. Representative data are shown from 2 independent experiments.

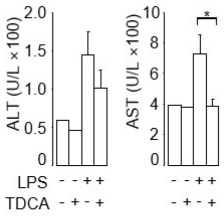

Figure S2. Blood ALT and AST levels in mice at 48 h after LPS injection. n=4, 4, 16 and 11 for the PBS+PBS, PBS+TDCA, LPS+PBS and LPS+TDCA groups, respectively. *, p<0.05 by the Student's two-tailed *t*-test. Data shown are pooled from 3 independent experiments with 1~3 mice per experiment.

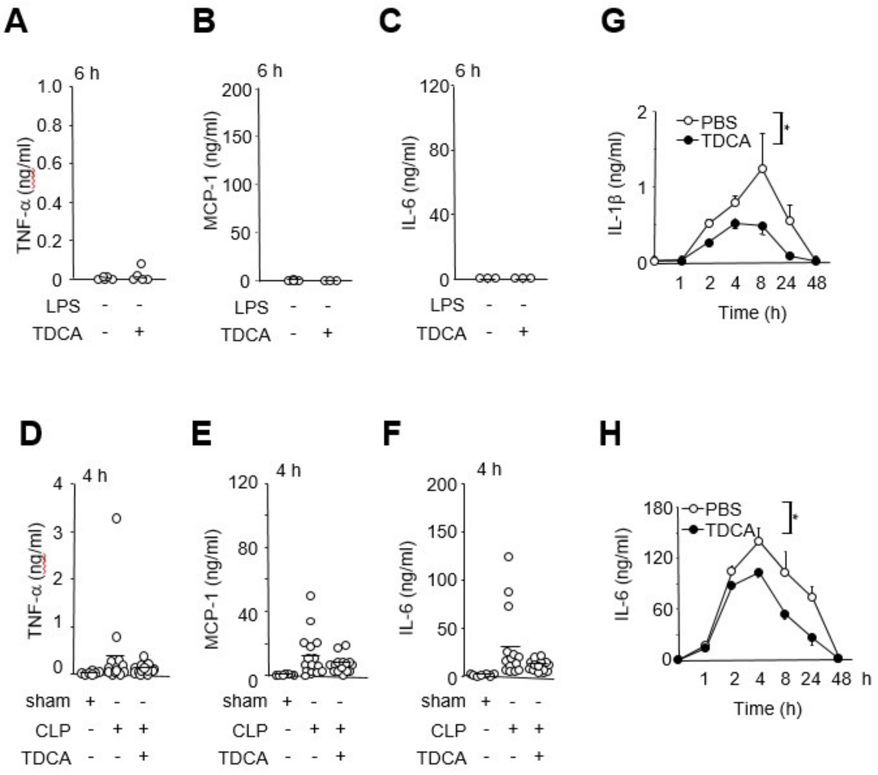

Figure S3. Pro-inflammatory cytokine production in B6 mice. (A, B and C) The blood levels of cytokines in mice at 6 h after TDCA infusion. $p > 0.05$ via the two-tailed Student's *t*-test. (D, E and F) The blood levels of cytokines of B6 mice at 4 h after CLP + PBS infusion or CLP + TDCA infusion. $p > 0.05$ via the two-tailed Student's *t*-test. (G) Plasma concentration of IL-1β at 48 h after LPS injection (n=8 (PBS or TDCA) for 0, 1, 2, 4 and 8 h; n=5 (PBS) or n=6 (TDCA) for 24 h, n=3 (PBS) or n=5 (TDCA) for 48 h). (H) Plasma concentration of IL-6 in LPS injection settings. The same sets of mice were used as in (G). *, $p<0.05$ via the Student's two-tailed *t*-test. Data shown are pooled from 4 independent experiments for LPS-injection setting or 6 independent experiment for CLP setting with 1~3 mice per experiment.

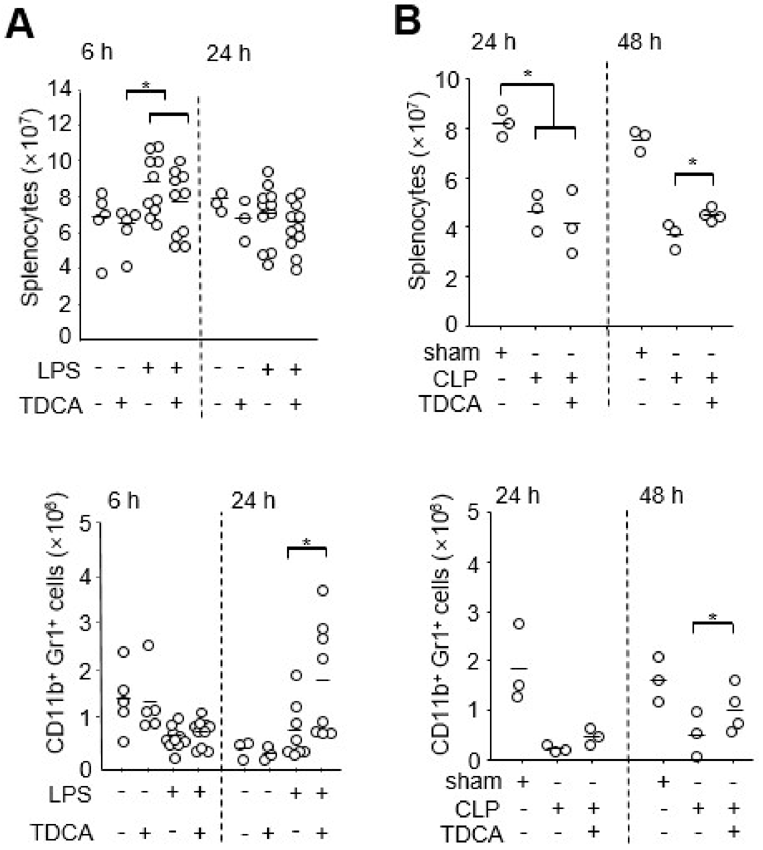

Figure S4. Numbers of total splenocytes and $CD11b^+Gr1^+$ splenocytes. (A) The numbers of total splenocytes (upper panel) and $CD11b^+Gr1^+$ splenocytes (lower panel) at 6 h or 24 h after LPS injection. (B) The numbers of total splenocytes (upper panel) and $CD11b^+Gr1^+$ splenocytes (lower panel) at 24 h or 48 h after CLP. *, $p<0.05$ via the Student's two-tailed *t*-test. Data shown are pooled from 5 independent experiments for LPS-injection setting or 3 independent experiment for CLP setting with 1~3 mice per experiment.

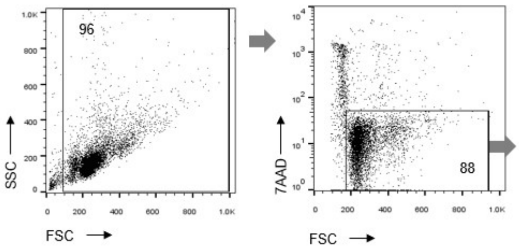

Figure S5. Gating strategies for the FACS analysis used for Fig. 2C.

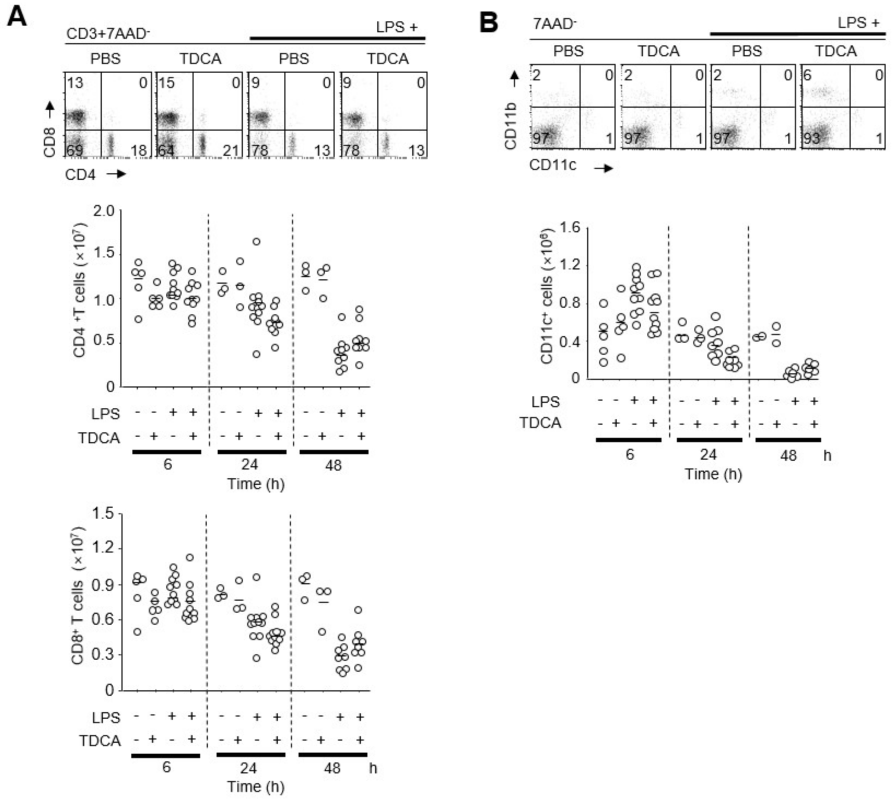

Figure S6. Numbers of splenic T cells and CD11c$^+$ cells after LPS $\pm$ TDCA treatment. (A) Representative FACS plots showing CD4$^+$ or CD8$^+$ T cells (upper panel) and the number of T cells after LPS injection (middle and lower panel). (B) Representative FACS plots showing CD11b$^+$ or CD11c$^+$ cells (upper panel) and scatter plot indicating the absolute number of CD11c$^+$ cells in the spleen (lower panel). The symbols in LPS + PBS or LPS + TDCA group, indicate individual mice from 5 independent experiments (for 6 h and 24 h) or 4 independent experiments (for 48 h). For PBS+PBS or PBS+ TDCA group, 2~3 independent experiments with one mouse per experiments. The bars in the middle of the circles indicate mean values. The thin bars and thick bars on the representative FACS plot indicate the gates for the FACS analysis and the LPS injection setting, respectively. $p > 0.05$ via the two-tailed Student's *t*-test.

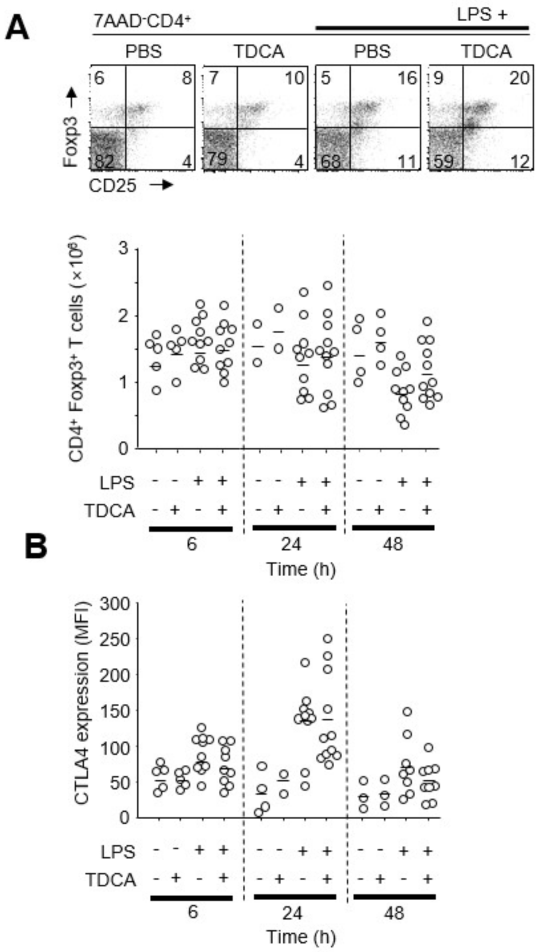

Figure S7. Number of splenic CD4^{+} CD25^{+}Foxp3^{+} T$_{reg}$ cells and CTLA4 expression on CD4^{+}CD25^{+}Foxp3^{+} T cells. (A) Representative FACS plots indicating the percentage of CD4^{+}CD25^{+}Foxp3^{+} T cells (upper panel) and number of CD4^{+} CD25^{+}Foxp3^{+} T cells after LPS injection (lower panel). The thin bars and thick bars indicate the gates for the FACS analysis and the LPS injection setting, respectively. (B) Scatter plot indicating the mean fluorescence intensity (MFI) of the CTLA4 expression on CD4^{+} CD25^{+}Foxp3^{+} T cells. The symbols indicate individual mice from 4 independent experiments (6 h and 24 h) or 5 independent experiments (48 h). The bars in the middle of the circles indicate mean values. Time (h) denotes the interval between LPS injection and FACS analysis. $p > 0.05$ via the two-tailed Student's *t*-test.

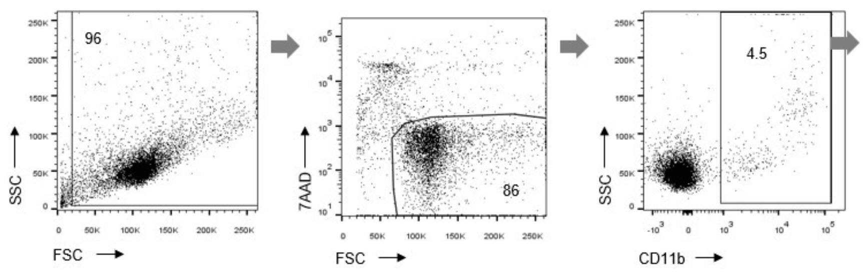

Figure S8. Gating strategies for the FACS analysis used for Fig. 2D.

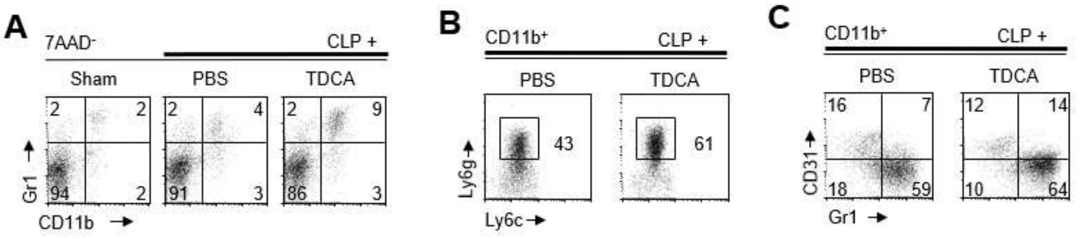

Figure S9. The surface phenotype of splenic CD11b$^+$ cells increased in CLP settings. (A) Representative FACS plot of splenic CD11b$^+$Gr1$^+$ cells in CLP settings. (B) The expression of Ly6c, Ly6g on CD11b$^+$ cells from spleen of mice in the CLP setting. (C) The expression of CD31 on CD11b$^+$Gr1$^+$ cells from spleen of mice in the CLP setting. Representative data from 3 independent experiments (A and B) and 2 experiments (C) are shown. The thin bars and thick bars on the representative FACS plot indicate the gates for the FACS analysis and the CLP setting, respectively.

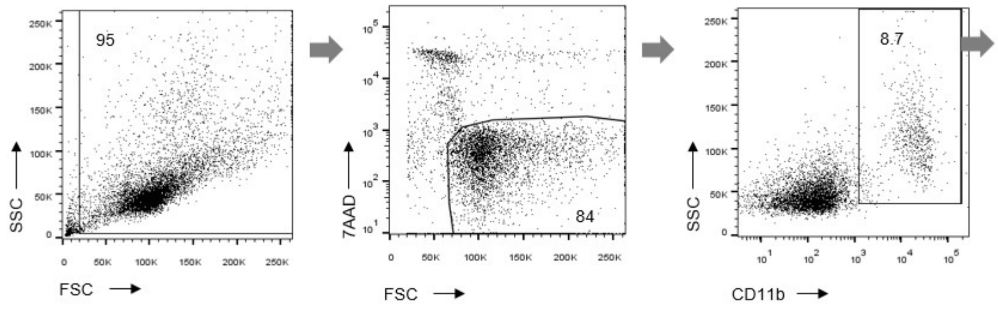

Figure S10. Gating strategies for the FACS analysis used for Fig. 2E.

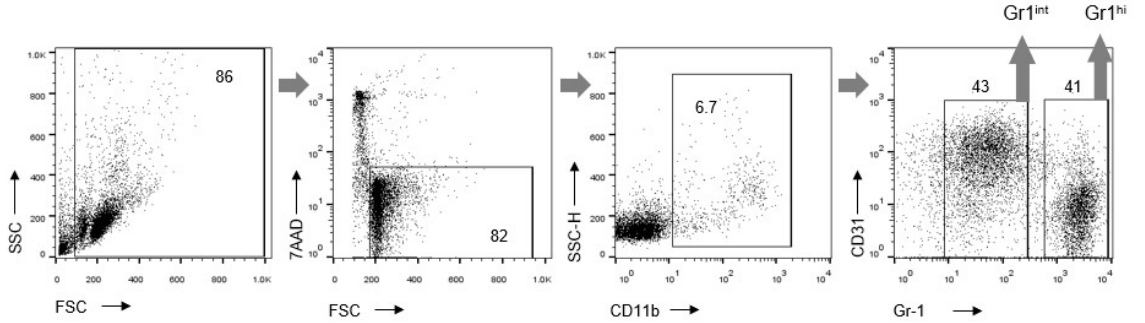

Figure S11. Gating strategies for the FACS analysis used for Fig. 2F.

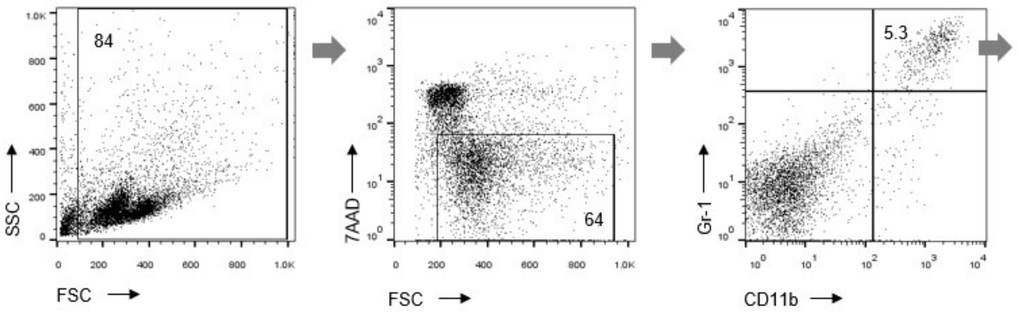

Figure S12. Gating strategies for the FACS analysis used for Fig. 2G.

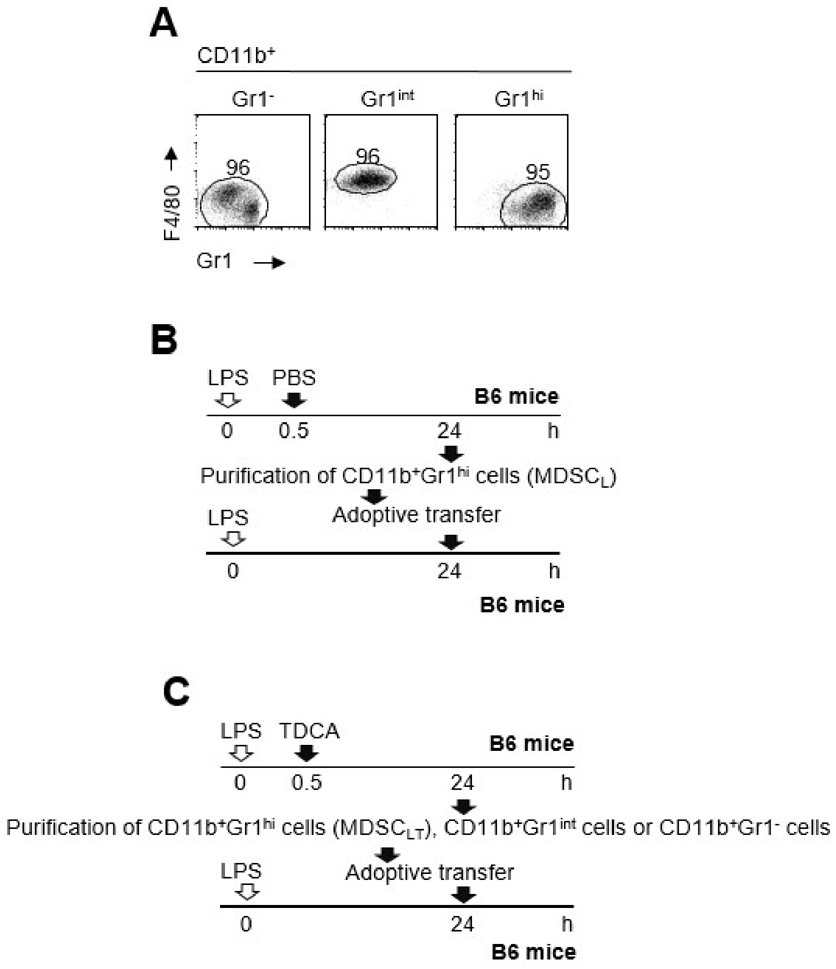

Figure S13. Representative FACS plots of the cells used in adoptive transfer and schematic timelines for adoptive transfer experiments. (A) Representative FACS plots indicating the purities of FACS-sorted cells used for adoptive transfer. The splenocytes were separated into 3 distinct groups of cells: $CD11b^+Gr1^{hi}$, $CD11b^+Gr1^{int}$ and $CD11b^+Gr1^-$. (B) The schematic timeline for the adoptive transfer of $CD11b^+Gr1^{hi}$ cells purified from the LPS+PBS group ($MDSC_L$) into B6 mice. (C) The schematic timeline for the adoptive transfer of $CD11b^+Gr1^{hi,\ int\ or\ -}$ cells purified from the LPS+TDCA group into B6 mice.

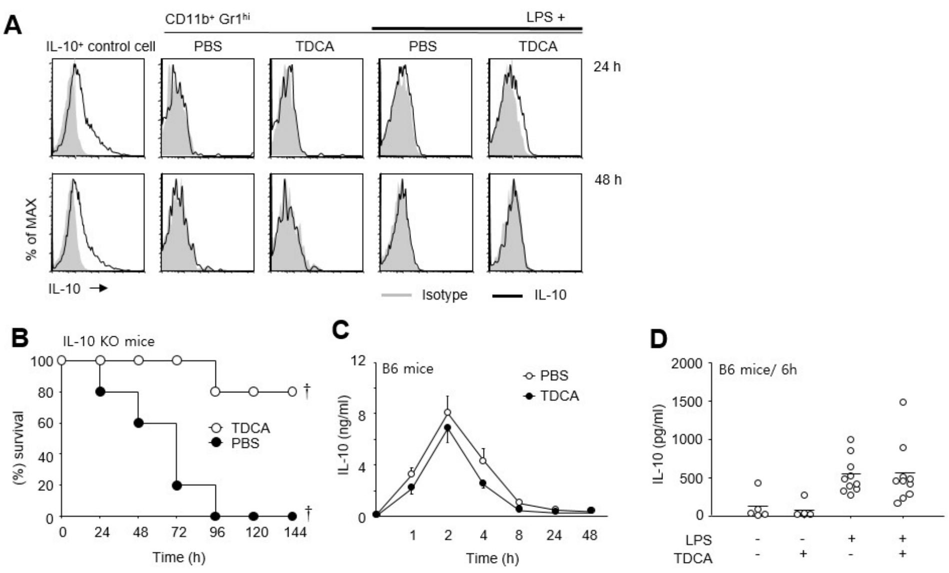

Figure S14. IL-10-independent protection of septic mice with TDCA. (A) IL-10 production by splenic CD11b$^+$Gr1^{hi} cells determined by FACS at 24 or 48 h after LPS injection. IL-10-producing MICK-2 cells were used as a positive control for IL-10 staining. The thin bar and thick bar on the representative FACS plot indicate the CD11b$^+$Gr1^{hi} gates and the LPS injection setting, respectively. Representative FACS plots of 3 independent experiments. (B) Survival of IL-10 KO mice under septic conditions following treatment with TDCA (n = 5) or PBS (n = 5) i.v. at 30 min after LPS injection. † indicates p<0.05 using the Kaplan-Meier survival risk assessment. (C) The time kinetics of IL-10 production in mice under septic conditions after treatment with PBS or TDCA (p >0.05, n=8 for 0 ~ 8 h, n=5 (PBS) or n=6 (TDCA) for 24 h, n=3 (PBS) or n=5 (TDCA) for 48 h. (D) IL-10 production in B6 mice at 6 h after LPS injection following treatment with TDCA or PBS (4 independent experiments with 1~3 mice per experiment). There was no statistically significant difference between the LPS + TDCA and LPS + PBS groups (p>0.05 via the Student's *t*-test). Data shown are pooled from 2 independent experiments with 2~4 mice per experiment otherwise denoted.

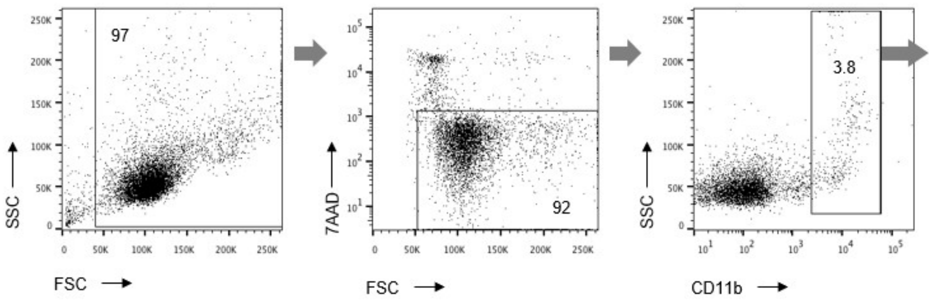

Figure S15. Gating strategies for the FACS analysis used for Fig. 5F.

Table S1. Primer sets, and PCR conditions used.

Target gene	Sequence	*Cycle
Arginase-1	Forward: CAGAGTATGACGTGAGAGACCAC<br>Reverse: CAGCTTGTCTACTTCAGTCATGGAG	94ºC for 15 sec<br>62.5ºC for 15 sec<br>72ºC for 30 sec<br>35 cycles
Prok2	Forward: TGCTGTGCTGTCAGTATCTGGGTT<br>Reverse: TTCGCCCTTCTTCTTTCCTGCCTT	94ºC for 15 sec<br>64.5ºC for 15 sec<br>72ºC for 30 sec<br>35 cycles

*All cycles were preceded by pre-denaturation at 94ºC for 2 min.
